# Reservoir quality and its controlling diagenetic factors in the Bentiu Formation, Northeastern Muglad Basin, Sudan

**DOI:** 10.1038/s41598-021-97994-x

**Published:** 2021-09-16

**Authors:** Yousif M. Makeen, Xuanlong Shan, Mutari Lawal, Habeeb A. Ayinla, Siyuan Su, Nura Abdulmumini Yelwa, Ye Liang, Ndip Edwin Ayuk, Xianli Du

**Affiliations:** 1 College of Earth Sciences, Jilin University, Changchun, 130061 China; 2 Department of Geology, University of Malaya, 50603 Kuala Lumpur, Malaysia; 3 Department of Geology, Usmanu Danfodiyo University, P. M. B 2346, Sokoto, Nigeria; 4 Department of Geology, Federal University Lokoja, P. M. B 1154, Lokoja, Nigeria; 5 Jiangxi Engineering Laboratory on Radioactive Geoscience and Big Data Technology, East China University of Technology, Jiangxi, China; 6 Pan African University-Life and Earth Science Institute, University of Ibadan, Ibadan, Nigeria; 7 School of Earth Sciences, Northeast Petroleum University, Daqing, 163318 China

**Keywords:** Energy and society, Environmental sciences, Energy science and technology

## Abstract

The Abu Gabra and Bentiu formations are widely distributed within the interior Muglad Basin. Recently, much attention has been paid to study, evaluate and characterize the Abu Gabra Formation as a proven reservoir in Muglad Basin. However, few studies have been documented on the Bentiu Formation which is the main oil/gas reservoir within the basin. Therefore, 33 core samples of the Great Moga and Keyi oilfields (NE Muglad Basin) were selected to characterize the Bentiu Formation reservoir using sedimentological and petrophysical analyses. The aim of the study is to de-risk exploration activities and improve success rate. Compositional and textural analyses revealed two main facies groups: coarse to-medium grained sandstone (braided channel deposits) and fine grained sandstone (floodplain and crevasse splay channel deposits). The coarse to-medium grained sandstone has porosity and permeability values within the range of 19.6% to 32.0% and 1825.6 mD to 8358.0 mD respectively. On the other hand, the fine grained clay-rich facies displays poor reservoir quality as indicated by porosity and permeability ranging from 1.0 to 6.0% and 2.5 to 10.0 mD respectively. A number of varied processes were identified controlling the reservoir quality of the studies samples. Porosity and permeability were enhanced by the dissolution of feldspars and micas, while presence of detrital clays, kaolinite precipitation, iron oxides precipitation, siderite, quartz overgrowths and pyrite cement played negative role on the reservoir quality. Intensity of the observed quartz overgrowth increases with burial depth. At great depths, a variability in grain contact types are recorded suggesting conditions of moderate to-high compactions. Furthermore, scanning electron microscopy revealed presence of micropores which have the tendency of affecting the fluid flow properties in the Bentiu Formation sandstone. These evidences indicate that the Bentiu Formation petroleum reservoir quality is primarily inhibited by grain size, total clay content, compaction and cementation. Thus, special attention should be paid to these inhibiting factors to reduce risk in petroleum exploration within the area.

## Introduction

**Figure 1:**
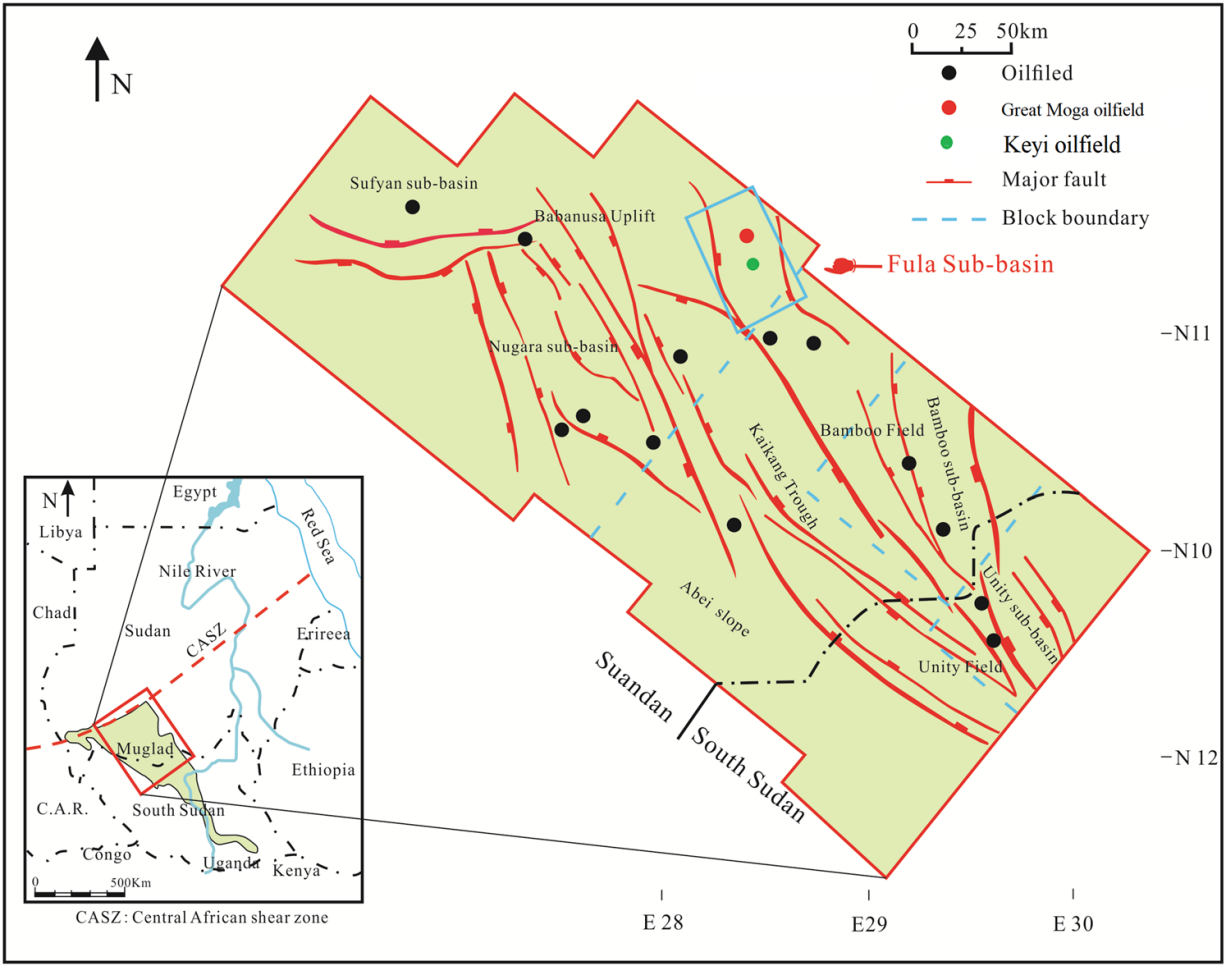
Location map showing the Great Moga and Keyi oilfields as well as the main oilfields discovered, major fault and block boundary, Fula Sub-Basin, Muglad Basin, Sudan (created from information in reference^1,2^ using CorelDRAW Graphics Suite 2018 v20.0.0.633 https://www.corel.com/cn).

The Great Moga together with Keyi oilfields of the Fula sub-basin are located in the northeastern region of the Muglad Basin (Fig. 1). The Fula sub-basin is about 120 km long and up to 40 km in width, while the Muglad Basin is about 120,000 km^2^ covering republic of Sudan and South Sudan (Fig. 1). It is believed to be the main hydrocarbon province in the two countries^1–3^. The petroliferous Muglad basin is an intracontinental rift basins whose formation, characteristics and hydrocarbon potential typify a major member of the petroliferous basins within Western and Central African shear zone formed in the Early Cretaceous as a result of opening of the South Atlantic^1–4^.

**Figure 2:**
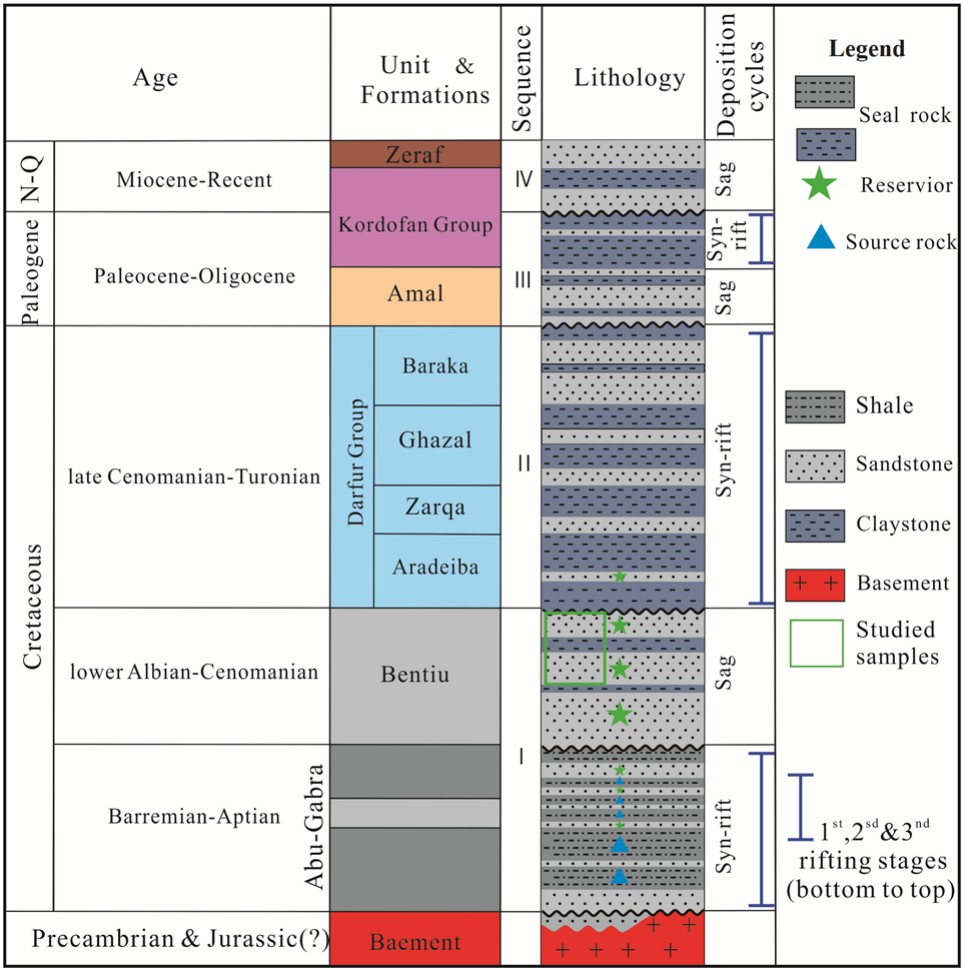
Main stratigraphic column of the Fula Sub-Basin. It compares sediments succession from Late Jurassic/Early Cretaceous–Quaternary with 4 succession (I-IV) separated by unconformities (created from information in reference^1,2^ using CorelDRAW Graphics Suite 2018 v20.0.0.633 https://www.corel.com/cn).

**Figure 3:**
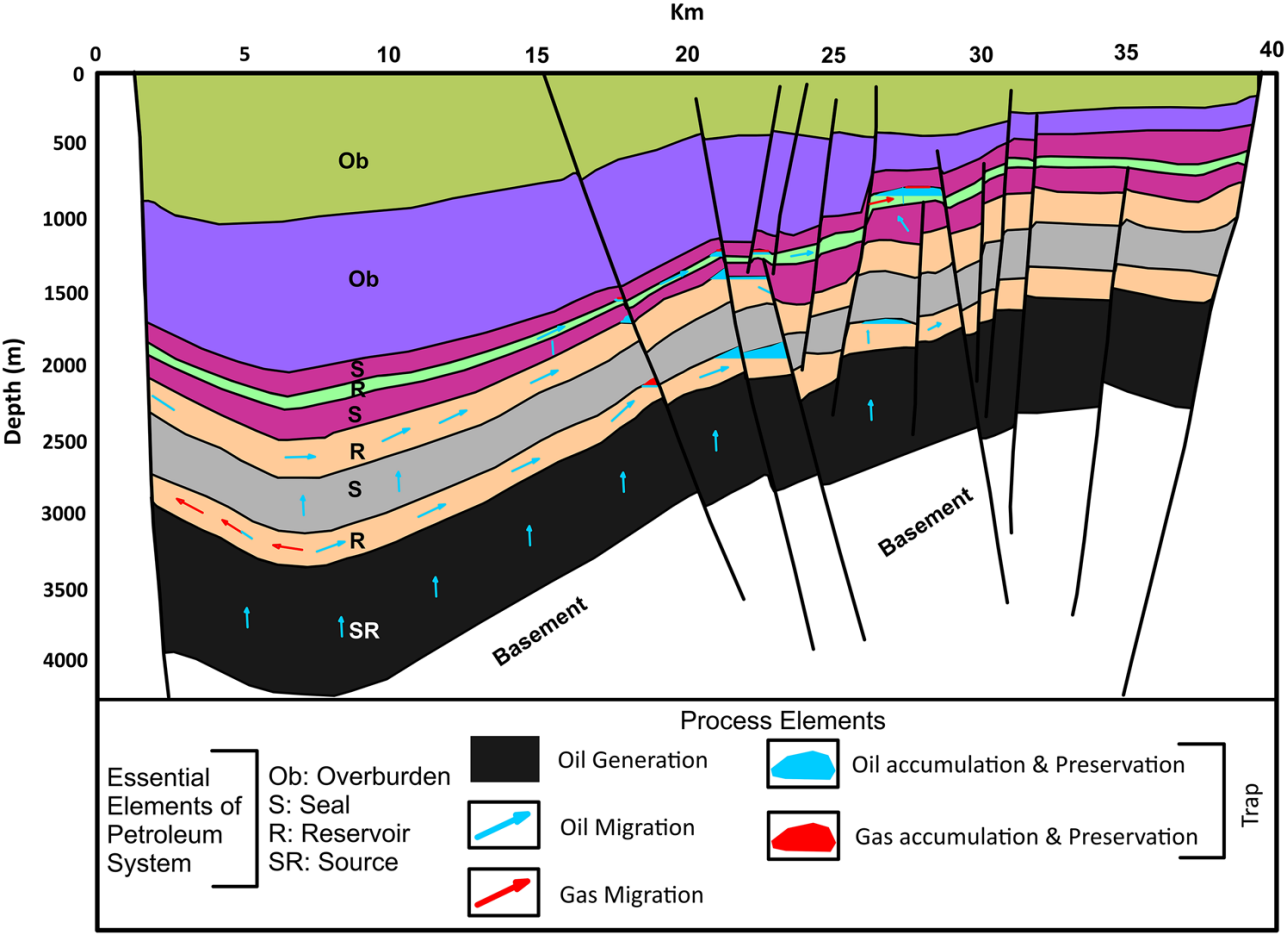
Petroleum system elements and processes of the Fula Sub-Basin with significant accumulation of oil and gas. (created from information in reference^11^ using CorelDRAW Graphics Suite 2018 v20.0.0.633 https://www.corel.com/cn).

Abu Gabra and the Bentiu formations (Figs. 2, 3) are widely distributed within the interior Muglad Basin, serving as source rock and reservoir respectively^4,5^. Source rock characteristics and hydrocarbon generation potentials of the Abu Gabra Formation have been studied by^1,2,6–8^. Like other Cretaceous source rocks around the world, especially Persian Gulf in the Middle East, Mediterranean, Gulf Coast of the USA and China, that have been suggested to have relatively good hydrocarbon prospects with Type I to III kerogens^1–4^. Authors^1,6–8^ reported that, the Lower Cretaceous shales and claystones of the Abu Gabra Formations represent the primary source rocks within the Muglad Basin. These are mainly characterized by Type I kerogen with very good to excellent hydrocarbon generation potentials. Thermal maturity assessment determined using vitrinite reflectance, biomarker parameters, pyrolysis gas chromatography and production index (PI) data indicated that the source rocks have reached the oil generation window^1,2,8,9^. Furthermore, an integrated trace/major elements and biomarkers study revealed suboxic to anoxic depositional conditions which favored preservation of organic matter in the Abu Gabra Formation^8,9^. Apart from the aforementioned studies, burial histories as well as petroleum generation modeling of the Abu Gabra Formations have been carried out^10^.

However, only little studies have been conducted on the Bentiu Formation despite being the major oil and gas reservoirs in the Muglad Basin. Previous research on the formation focused mainly on its depositional environment and general reservoir properties without thorough investigation on its mineral composition and diagenetic reservoir quality^12–14^. Globally, a number of methods have been used to access reservoir quality, depositional modelling and diagenetic effect of world class reservoirs for optimization of their potentials^4,13–17^. Out of which, it is well known that sedimentology and diagenesis are key to reservoir properties accessment^14–17^. Therefore, there is a need for more investigations on these aspects to guide on future hydrocarbon exploration in the Muglad Basin. To do this, based on available data, samples from the Great Moga and Keyi oilfields of the Fula sub-basin (northeast portion of Muglad Basin) are used for this research.

Within the Fula Sub-Basin, clayey shales of the Abu Gabra Formation are very good source rocks. The Abu Gabra Sandstones, the thick Bentiu Formation sandstones as well as Aradeiba, Zarga and Ghazal sandstones (Darfur Group) are the major reservoir rocks, while claystones of Darfur Group serve as regional seals^3,11,18^. Thus, the petroleum systems of Fula Sub-Basin are considered to be effective and capable of preserving significant accumulation of oil and gas (Fig. 3). The purpose of this research is to emphasis the diagenetic processes controlling the reservoir quality of the Bentiu Formation. To achieve this, sedimentological, petrographical and petrophysical analyses were performed on some selected core samples. These involve detailed sedimentological and petrographical descriptions of the core intervals stating facies type and their associations to determine their depositional environments. In addition, scanning electron microscopy (SEM) was used to have better understanding of the different pores types. The effect of diagenesis on the reservoir quality was investigated to identify the major controls of porosity and permeability on the studied reservoir facies. Other important information presented in this study include the basin evolution and sediment transportation processes. Moreover, heavy mineral analyses were also performed on selected core samples. Results of this study can be utilized to enhance the chances of success in the exploration activities within the region and other similar area around the world.

## Geological setting

The late Jurassic marks the beginning of rifting in Sudan resulting from right-lateral motion of Central African Shear Zone (CASZ) during the opening of the Southern Atlantic Ocean^2,11,18^. The CASZ bifurcates north central Africa through Benue Trough of Nigeria, to Bornu basin up till Sudan. Six main sedimentary basins (Muglad, Baggara, Melut, Khartoum, Blue Nile and Atbara) in Sudan were devolved as a result of this event^2,11,18^. The Sudanese basins as well as Benue Trough, Bongor, Doba and Ngaoundere basins are similar in terms of sediment distribution and evolution^2,11,18^. Being products of a similar tectonic event; each of the basins mentioned exhibit some unique characteristics that are results of local influences.

The Muglad Basin is among the largest rifted basins in Northern Africa which is composed of bulk petroleum accumulation as typified in Fula, Heglig as well as Unity oil field^2,11,19^. Seismic evidence shows that the maximum sediment thickness within the Muglad Basin is about 13 km^4^. The stratigraphy of Fula Sub-Basin is as shown in Fig. 2. It is made up of about 8200 km succession of Late Jurassic/Early Cretaceous—Quaternary non-marine sediments. Based on seismic interpretation and drilled well data, the stratigraphic column has been subdivided into four successions, (I–IV; Fig. 2) bounded by unconformities^2,3,11,20^. Within the Fula sub basin, sediments overlies on quartzites, schist, granitic and granodioritic gneisses rocks (Fig. 2). These granitic and granodioritic gneisses have been dated 540 ± 40 Ma using isotopic method^4^. Three rifting stages have been recognized in the Fula sub basin on account of dextral dynamism of the CASZ^5^. The rifting initiation took place during Early Cretaceous to Albian time that resulted in deposition of the Abu Gabra and Bentiu Formationsn^2,3,20^. The Abu Gabra Formation is made up of thick shale, sandstone and claystone units, deposited mainly in a suboxic to anoxic lacustrine environment which received large quantities of algae, bacterial and amorphous organic material^1,4^. Abu Gabra Formation has high total organic carbon (average 4.5 wt.%) and hydrogen index (average 640 mg HC/g TOC) indicating Type I kerogen^1,7,8^. In this manner, Abu Gabra Formation might be an important oils source within the Bentiu Formation reservoirs. During Early Albian through Cenomanian time, Bentiu Formation was deposited within braided and meandering stream environments^3,4^. Based on previous geologic reports, we have lower Bentiu Formation and upper Bentiu Formation^10,12^. The lower part is characterized by fining upward sequence of channel sandstone interbedded with floodplain facies, whereas the upper part is composed of thick sandstones intercalated with thin layers of mudstones^4,10,12^ deposited under a braided stream setting.

The second rifting (Fig. 2) occurred from the Late Cenomanian to Turonian during which the Darfur Group was deposited^4,19^, represented by four formations: Aradeiba, Zarga, Ghazal and Baraka Formations as shown in Fig. 2. Aradeiba Formation is composed of bulky mudstones intercalated with thin sandstones. Conformably overlying the Aradeiba lies the Zarga Formation which was deposited by a fluvio-deltaic channels within a lacustrine environment. It is recognized by an upward transition from sandstone (basal) to mudstone (top) units^4^. The Ghazal Formation has a lithological characteristic like the Zarga Formation, equally comprising of interbedded sandstone and mudstone sequences^4^. The Baraka Formation appear as the topmost strata of the Darfur Group made up of sandstones interbedded with thin mudstones, deposited under fluvial to alluvial fan environment^4,5^.

Towards the end of the second rifting, the Early Paleogene Amal Formation, containing mainly intermediate to coarse grained sandstone sequences, was deposited as braided flow and alluvial fan^4^. Third rifting resulted in the formation of Kordofan Group between Late Eocene to Miocene period^4^. The Zeraf Formation, which is singularize by thick sandstone interbedded with thin clays unconformably overlies the Kordofan Group. The sediments of this formation are products of braided stream and alluvial fan^3–5^.

## Data and methods

Sedimentological and petrographical analyses of five cores (core-1–core-5) from Moga 6 well and four cores from Moga 26 (core-1–core-4) were carried out. In all, a total number of 33 samples was selected for lithofacies studies. Table 1 shows a compendium of the analyzed samples, their depth intervals, facies types, analyses performed and total length of these cores. Five sedimentological techniques (see Table 1) were employed to investigate the core samples. The procedures for the combined techniques are briefly explained in the following subsections:

**Table 1:** Compendium of analyzed core samples from Moga 26, Moga 6 and Keyi 4 wells in the Fula Sub-basin Muglad Basin, Sudan.

Well name	Core no	Intervals (m)	Analyses performed				
			GS	TS	SEM	XRD	HM
**Moga 26**	Core 1	797.07–798.58	2	2	1	1	1
	Core 2	799.00–801.81	3	3	1	1	1
	Core 3	802.50–803.99	2	3	1	2	2
	Core 4	806.38–807.23	1	2	2	–	1
**Total**		**6.73**	**8**	**10**	**5**	**4**	**5**
**Moga 6**	Core 1	860.00–861.70	2	3	3	2	2
	Core 2	895.20–897.47	1	3	1	4	1
	Core 3	899.00–900.30	2	2	2	1	1
	Core 4	902.00–903.00	1	2	2	1	1
	Core 5	906.50–910.93	2	4	1	4	1
**Total**		**10.67**	**16**	**14**	**9**	**12**	**6**
**Keyi 4**	Core 1	1510.27–1513.99	2	3	2	2	1
	Core 2	1513.99–1516.37	2	1	–	–	1
	Core 3	1688.32–1695.70	4	5	2	2	1
**Total**		**13.52**	**8**	**9**	**4**	**4**	**3**

Abbreviations for Analyses performed: GS: Grain-size Analysis; TS: Thin Section Analysis; SEM:Scanning Electron Microscope Analysis; XRD: X-Ray Diffraction Analysis of the < 2 and HM: Heavy Minerals Analysis.

**Figure 4:**
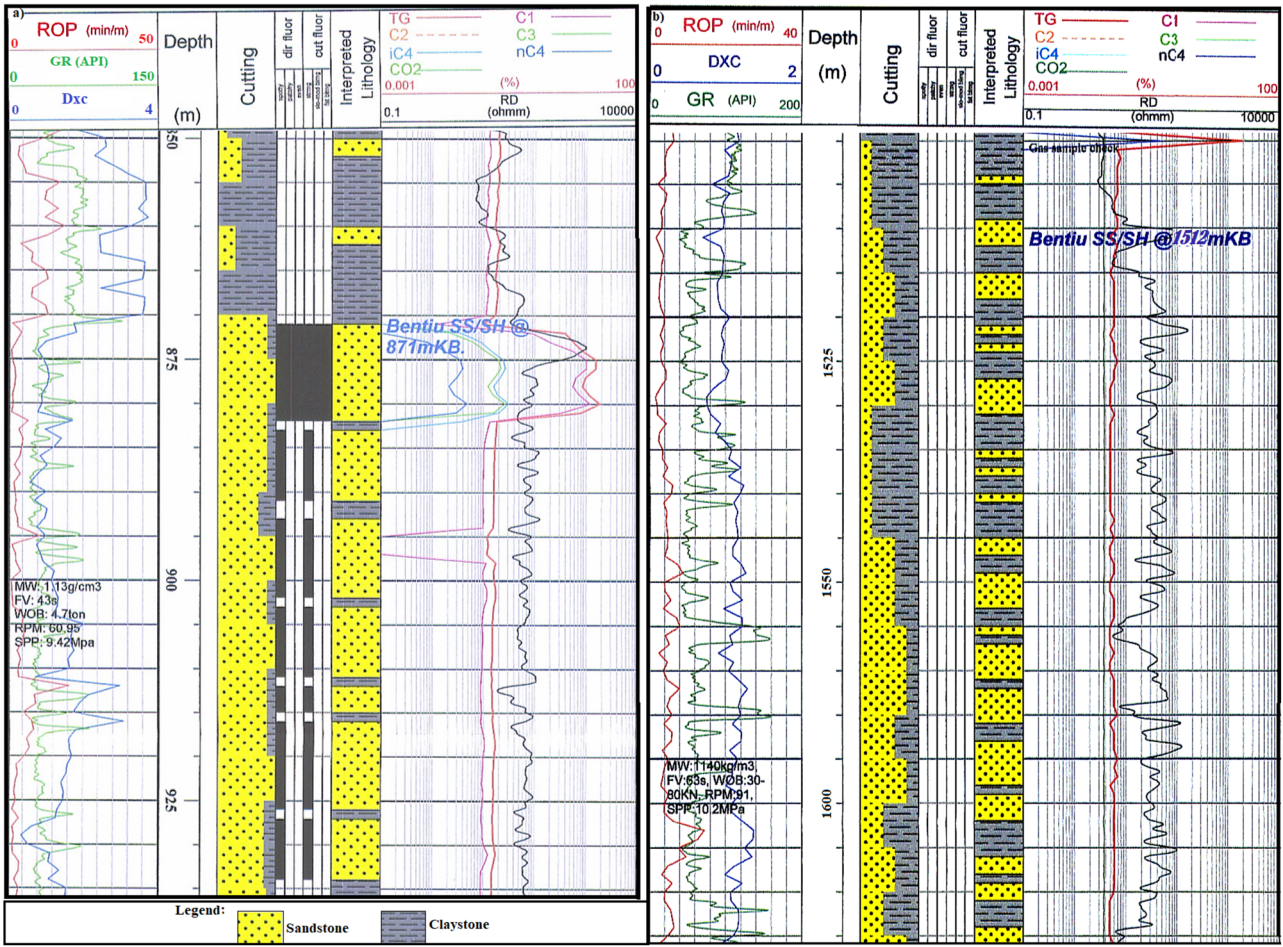
Well log response of the Bentiu Formation in the Great Moga (**a**) and Keyi (**b**) fields showing the results of the wire line logs and lithology (sandstone/claystone) within Bentiu Formation. A suite of measurements includes rate of penetration (ROP), gamma ray (GR) and D-exponent (Dxc). The gamma ray response distinguishes the low gamma ray value of sand from the higher value of shale. The next column (depth track) indicates the depth at which the measurement was made.

Facies determination was undertaken using 6.73 m, 10.79 m and 13.52 core samples from well Moga 62, well Moga 6 and well Keyi 4 respectively. These were sedimentologically analyzed and interpolated against the core gamma at a vertical scale of 1:20 (Fig. 4). Cores number and depth interval are shown in Table 1. The lithologic characteristics of the core were examined both macroscopically (naked eye) and microscopically (hand lens and binocular microscope). Rock types are classified based on their specific characteristic (e.g. mudstone, sandstone) and were further described according to their colour, composition, grain-size distribution, sedimentary structures and textures. Interpretation of the depositional environment is based on observations and thorough assessment of all available evidences (e.g. interpretation of the wire line logs of the studied well).

Grain size analysis was performed, using a total number of sixteen core samples drawn mostly from the different facies types (Table 1). Before the sieve analysis, two preparatory methods were applied in order to disintegrate the sandstone samples into individual grains without breaking or destroying them. Instrumental disintegration of the samples was pursued by addition of hydrogen peroxide solution (30% w/v). Then the samples were immersed in water and carefully mortared in a porcelain mortar and shacked by the shaker for about 48 h. Thereafter, the samples were oven-dried under 40 °C for about 24 h. This was followed by complete disintegration and dryness of the samples so as to subject them to mechanical wet sieving analysis. During the sieve analysis, 40 g of samples were weighed and analyzed by OCTAGON digital (00363) for 15 min under amplitude 4 with intermittent instrumental shaking. With the aid of brush and Ultrasonic, each grain size fraction was collected from the sieves into porcelain dishes. These fractions were oven dried at 40º C. After complete dryness their weights were recorded (Table 1).

Furthermore, 33 thin sections were made from selected core samples for petrographic description (see Table 1). To recognize porosity and analyze carbonate minerals, the samples were subjected to vacuum impregnation with a blue-dyed resin during thin section preparation, mixed with Alizarin Red-S and potassium ferric cyanide staining. The samples were further stained with some sodium cobalt nitrate solution in order to aid identification of the alkali feldspars. The thin sections were observed under polarized microscope at different magnifications. Optical properties of minerals such as colour, form, relief, pleochroism, angle of extinction, birefringence and twinning were used as basis for identifying minerals. Point counting was conducted using PETRLOG to determine the minerals percentages in each slide. Each thin section was point-counted (250–300 points) and the summary of the petrographic results are presented following Dott classification schemes for rocks^21^.

In this study, scanning electron microscopy (SEM) of the Bentiu Formation involve. The samples were initially treated with cold chloroform to clear out any hydrocarbon residues before being fixed onto standard aluminum SEM stubs using sputter aluminum tape. This experiment focuses on the pore geometry, composition and morphology of the main pore-filling authigenic minerals. Results from the SEM analysis are included in the “clay minerals analysis” section. Less than two (< 2) microns of 20 clay fractions were examined under X-ray diffraction (XRD) approach following the method introduced by Moore and Reynolds^21^. Explanations were made by comparing observations from this study with previous studied^3,22^. Heavy minerals analysis was performed on eleven core samples (Table 1). This follows the identification techniques of Hubert^23^. Relative porosity and permeability to oil and water were calculated as a function of water saturation using the techniques described by Jones and Roszelle^14^ and Makeen et al.^18^.

## Results and interpretations

### Facies characterization

**Figure 5:**
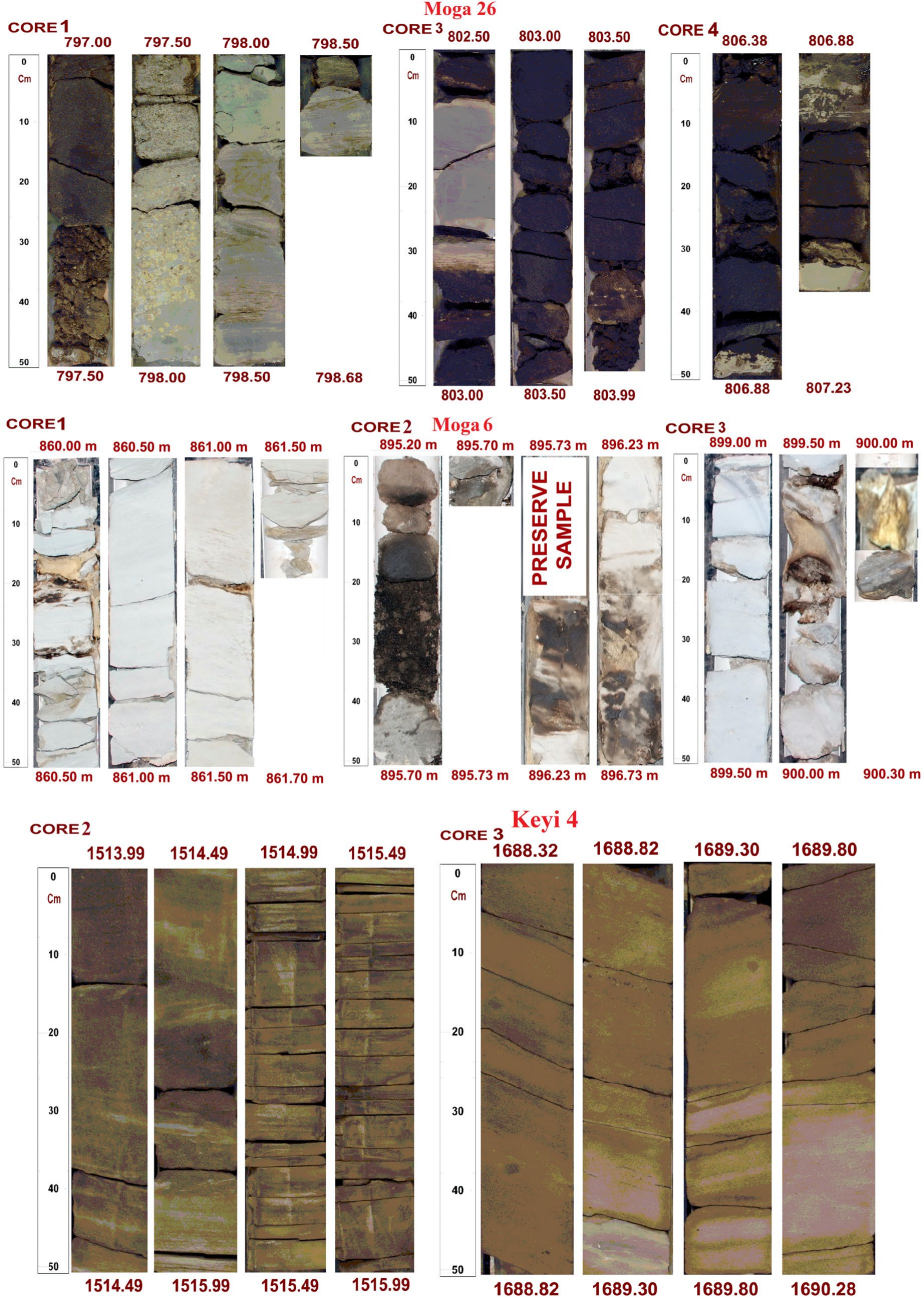
Conventional cores photos showing the studied core form different wells. Eight (8) different major lithofacies types have been recognized form these photos (Tables 2 and 4).

Observations of the conventional cores (e.g. Fig. 5) revealed eight types of lithofacies. Total thickness, percentage and simple interpretation of these lithofacies are shown in Table 2. All the facies recognized here follows Miall classification scheme^24–26^. Detail descriptions of these lithofacies are as follows:

**Table 2:** Summary of the facies description presents in the studied core intervals.

Well	Facies code	Lithofacies	Total thickness (m)	Percentage (%)	Interpretation
**Moga 26**	Sm	Massive sandstone	4.10	60.92	Rapid sedimentation (high-discharge event)
	Sxl	Cross–Laminated Sandstone	0.25	3.71	Sloping surface (ripple marks) from high-energy fluvial origin
	Sm/ Sxl	Massive/Cross–Laminated sandstone	0.80	11.89	Linguoids, transverse bars, sand waves (fluvial channel or delta mouth bar)
	Fm	Massive to Blocky mudstone & siltstone	0.25	3.71	Proximal floodplain to more distal deposits
	Sco	Conglomeratic Sandstone	0.53	7.88	Channel lag deposits (high energy)
	Sr	Ripple Laminated Sandstone	0.35	5.20	Channel abandonment facies (Lower flow regime)
	Fl	Fine laminated sandstone	0.45	6.68	Over bank (deposition from suspension and weak traction current)
**Total**			**6.73**	**100.00**	
**Moga 6**	Sm	Massive sandstone	8.82	81.74	Rapid sedimentation (high-discharge event)
	Sr	Ripple Laminated Sandstone	0.91	8.43	Lower flow regime
	Fm	Massive to Blocky mudstone & siltstone	0.73	6.77	Proximal floodplain to more distal deposits
	FI	Fine laminated sandstone	0.33	3.06	Over bank (deposition from suspension and weak traction current)
**Total**			**10.79**	**100.00**	
**Keyi 4**	Sm	Massive sandstone	5.50	40.70	Rapid sedimentation (high-discharge event)
	Sxl	Cross–Laminated Sandstone	3.78	28.0	Linguoids, transverse bars, sand waves (fluvial channel or delta mouth bar)
	Sxt	Trough cross-bedded sandstone	2.04	15.1	Dunes (fluvial channel or delta distributary channel)
	Sh	Horizontal laminated and parted sandstone	0.26		
	1.90	Planar bed flow (fluvial channel bar deposits or delta mouth bar deposits)			
	Sr	Ripple Laminated Sandstone	0.40	3.0	Lower flow regime
	FI	Fine laminated sandstone	1.54	11.4	Overbank or waning flood deposits or delta distal bar deposits
**Total**			**13.52**	**100**	

Facies codes (Sxl, Sm, Sr, Fm and Fl) according to Miall (1978, 1996 & 2006) classification.

#### Massive sandstone (Sm)

Massive sandstone (light grey to grayish) is the most dominant facies type observed (with high quantity) in all of the studied wells (Table 2). These facies are locally massive and have faint bedding planes. Its grains are very coarse lower to medium lower (vcL—mL), subrounded to rounded (SR-R), with moderate to well sorted particles (Table3). The porosity in these facies varies from good to very good (19.6% to 32%). These facies are believed to have developed from very rapid sedimentation like high discharge processes e.g., sheet floods.

**Table 3:** Grain size analysis result.

Moga 6: Depth = 861.20 m ; Lithofacies = Sr						Keyi 4: Depth = 1510.55; Lithofacies = Sm					
Size grade	Sieve opening (mic)	Phi (Ǿ)	Weight (gms.)	Weight %	Cumulative %	Size grade	Sieve Opening (mic)	Phi (Ǿ)	Weight (gms.)	Weight %	Cumulative %
Pebble	4000	− 2.00	0.00	0.00	0.00	Pebble	4000	− 2.00	0.86	2.15	2.15
Granule	2000	− 1.00	0.00	0.00	0.00	Granule	2000	− 1.00	5.01	12.53	14.68
V.C Sand	1000	0.00	0.00	0.00	0.00	V.C Sand	1000	0.00	10.88	27.20	41.88
C. Sand	710	0.50	0.00	0.00	0.00	C. Sand	710	0.50	7.38	18.45	60.33
	500	1.00	0.27	0.68	0.68		500	1.00	7.01	17.53	77.85
M. Sand	355	1.50	0.72	1.80	2.48	M. Sand	355	1.50	4.80	12.00	95.43
	250	2.00	3.59	8.98	11.45		250	2.00	2.23	5.58	97.83
F. Sand	180	2.50	12.04	30.10	41.55	F. Sand	180	2.50	0.96	2.40	98.58
	125	3.00	13.36	33.40	74.95		125	3.00	0.30	0.75	98.83
V.F. Sand	90	3.50	6.86	17.15	92.10	V.F. Sand	90	3.50	0.10	0.25	99.45
	63	4.00	2.08	5.20	97.30		63	4.00	0.25	0.63	99.45
Silt	45	4.50	0.38	0.95	98.25	Silt	45	4.50	0.00	0.00	99.4
	32	4.75	0.00	0.00	98.25		32	4.75	0.00	0.00	99.45
Pan	10 < 37	6.50	0.18	0.45	98.70	Pan	10 < 37	6.50	0.00	0.00	100.00
Sieve Loss			0.52	1.30	100.00	Sieve Loss			0.22	0.55	–

**Moga 6:Granulometric composition (**Sample Depth: 860.70 m; Total Weight: 40 g; Facies Code: Sr**)**.

Gravel: 0.00%.

Sand: 98.57%.

Mud: 1.42%.

**Sample description (**Fine sand, moderately sorted, near—symmetrical, leptokurtic).

Keyi 4: Granulometric composition (Sample Depth: 1510.55 m; Total Weight: 40 g; Facies Code: Sm).

Gravel: 14.76%.

Sand: 85.23%.

Mud: 0.00%.

**Sample description** (Coarse sand, poorly sorted, near—symmetrical, Mesokurtic).

#### Ripple laminated sandstone (Sr)

These are made up of grain sizes ranging from 0.7 to 1.0 mm, commonly light grey–grey, fine lower to fine upper (fL–fu) ripple laminated sandstone with sub-rounded to rounded shapes (Table 4). The facies were detected only in a few cores with percentage not exceeded 8.43%a (Table 2). Moreover, the grains are well sorted. The ripple structures observed in these facies are indicative of wave/current episodes in lower flow regime sedimentary depositional process. Hence, the rippled laminated sandstone can be elucidated as braided channel sediments, whereas the rippled marked siltstone can be interpreted as overbank deposits^3,26^.

**Table 4:** Statistical data of the analyzed core samples.

Well	Core no	Depth (m)	Facies code	Minimum diameter (mm)	Maximum diameter (mm)	Sieve loss (%)	Grain-size parameters			
							Grain size class	Sorting	Skewness	Kurtosis
**Moga 26**	**1**	797.07	Sm	0.09	1.00	1.40	Very Coarse	Well	**Near Symmetrical**	Leptokurtic
		797.60	Sco			1.50		Moderately		Mesokurtic
	**2**	799.03	Sm			_1.30_				Platykurtic
		800.25				_1.35_				Mesokurtic
		801.00				_1.20_				Leptokurtic
	**3**	802.80	Sr	0.70		_1.80_	Fine	Poorly		V.Platykurtic
		803.07	Sm/ Sxl			_1.50_	Coarse	Moderately		Platykurtic
		803.57				_0.90_				Mesokurtic
	**4**	806.42	Sm			_0.75_				Mesokurtic
		807.20	Sr	0.70		_1.95_	Fine			Leptokurtic
**Moga 6**	1	860.70	Sm	0.09	1.00	1.30	Coarse	Moderately	**Near Symmetrical**	Leptokurtic
		861.20				1.50				Mesokurtic
	2	897.10	Sm			1.10	Coarse	Poorly		
	3	899.35				1.48				Platykurtic
		899.83				0.97				Mesokurtic
	4	902.30				0.95	Very Coarse			V.Platykurtic
	5	908.00				0.73	Coarse	Moderately		Mesokurtic
		910.40				0.55		Poorly		
**Keyi 4**	**1**	1510.55	Sm	0.09	1.00	1.60	Very Coarse	Well	**Near Symmetrical**	Mesokurtic
		1511.55				1.50				Platykurtic
		1513.50	Sxl			1.20		Moderately		Mesokurtic
	**2**	1515.55	FI	0.07		1.42	Fine	Poorly		Leptokurtic
	**3**	1689.55	Sxl	0.09		1.09	Coarse	Moderately		V.Platykurtic
		1691.40	Sm			1.38				Platykurtic
		1692.00	FI	0.07		1.12	Fine	Poorly		Mesokurtic
		1693.00	Sxt	0.09		0.73	Coarse	Moderately		Platykurtic
		1695.45	Sm			1.30				Mesokurtic

#### Massive to blocky mudstone (Fm)

Massive to blocky mudstone facies are represented in a few cores, reaching a total thickness of 0.92 m (Table 2). They occur as grey–dark grey, massive blocky mudstone. These facies could be interpreted as deposits of overbank in a fluvial environment^3,25^.

#### Fine laminated sandstone (Fl)

Fine laminated sandstone facies can be observed in all the studied wells cores (Table 2). The colour is grey to dark-grey. This facies type is common in overbank areas and appear as sediments from weak traction currents consistent with Miall facies classification^24,25^.

#### Cross—laminated sandstone (Sxl)

This facies is well-distributed in Moga and Keyi areas with considerable quantities (Table 2). It comprises of medium grained (mL to mU) sandstone. The colour varies between grey to grey-yellowish with rounded and well sorted grains. Although, it has very good porosity (average 23.7%), some clay matrix, few carbonates and little iron oxide occurring as cementing material which may affect its reservoir quality. This facies is interpreted as linguoid, transverse bars or sand waves channelized deposit within a fluvial system or deltaic mouth bar settings.

#### Conglomeratic sandstone (Sco)

This grey very coarse-grained facies (vcU) is only present in well Moga 26 with a total thickness of 0.53 m (Table 2). The clasts are subangular—subrounded, poorly to moderately sorted grains that are embedded within a silty mudstone matrix. This facies is interpreted as products of relatively high energy fluvial channel or a deltaic mouth bar judging from its grain composition, sizes and shapes.

#### Trough cross-bedding sandstone (Sxt)

These facies are mostly found in Keyi area with total thickness of about 2.04 m (Table 2). It is a grey to yellowish colour, fine grained sandstones (fL–fU; 0.09 to1.0 mm). The grains are rounded, well sorted and has high porosity (average 26.5%). Some siliceous cement was observed, while oil-shows are present as dispersed dots. These facies are interpreted as products of scour fills, washout dunes or antidunes deposited in fluvial channel or distributary channel of a delta.

#### Horizontal laminated sandstone (Sh)

These facies also present in Keyi area with a total thickness not exceeding 0.26 m (Table 2). It is a grey to yellowish fine grained sandstone (fU). The grains are well sorted as well as rounded to well-rounded sediments deposited under fluvial system or deltaic mouth bars.

### Granulometric analysis

**Figure 6:**
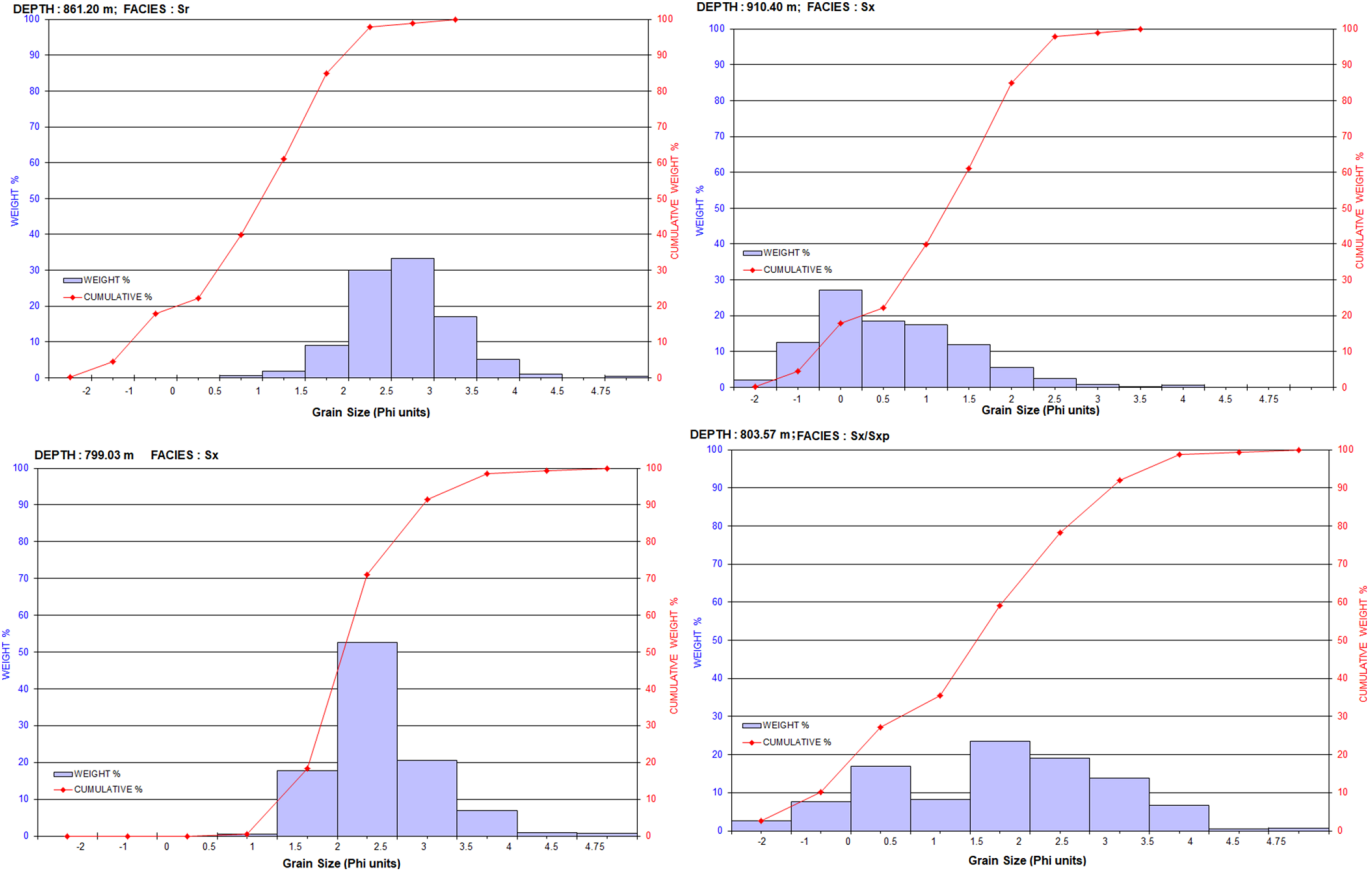
Representative, grain size and sieve analyses histograms showing cumulative weight percentage and grain size distribution.

The granulometric analysis was performed on some selected core samples in order to identify the potential sandy intervals, grain size distributions and characteristics. Grain size analysis is also a supportive tool for facies analysis. Representative grain size and sieve analyses data, besides the cumulative weight percentage plots, are presented in Fig. 6 and Table 3.

The grain-size analysis revealed that all the studied samples contain < 0.07 mm–1.00 mm fraction of the sand-size. Silt and clay size (< 0.063 mm) occur in minor quantities in all the studied samples. Furthermore, components of grain-size range between greater than 500Im and less than 90Im (< 0.07– > 0.5 mm) represent the dominant constituents in all of the studied samples. The granulometrical analysis indicated that two of the analyzed samples are moderately sorted, whereas the rest are poorly sorted. Table 4 summarizes the statistical data of the analyzed samples according to their facies types and association. In addition, petrographical analyses under the plane polarized microscope and the scanning electron microscope for the sandstone lithofacies types within the cored intervals allowed classifying these lithofacies into feldspathic arenite and subfeldspathic arenite (Table 5). Detail granulometric study including grain-size, sorting, shape, sorting and packing are described in facies characterization and discussion sections.

**Table 5:** Petrographic data for the studied samples.

Well	Depth (m)	Facies	Textural data					Rock name	Detrital mineralogy										Authigenic minerals					Porosity			Permeability (mD)
			Grain sorting	Grain contacts	Grain roundness	AV. Pore connectivity	Pore types		Poly crystalline Qtz. %	Monocrystalline Qtz. %	Total Qtz %	Lithic fragment. %	K-feldspar %	Plagioclase %	Micas %	Heavy minerals %	Clay matrix %	Calcite cement %	Siderite cement %	Qtz over growth %	Pyrite cement %	Total cements	Iron oxide cement %	Primary %	Secondary %	Total porosity%	
**Moga 26**	**797.07**	**Sm**	PS	PLC	SA-SR	Good	PBP SWP SBP	Subfeldspathic Arenite	11.7	30.5	42.2	1.0	11.0	3.4	0.5	1.5	2.5	*	2.5	0.2	1.8	7.0	3.4	27.0	3.0	30.0	6359.0
	**797.60**	**Fl**	VPS	PLC	SA-SR	Poor		Sublithic Arenite	11.0	13.5	25.5	9.5	2.0	1.7	*	0.5	45.0	*	6.5	0.1	2.9	54.5	1.3	4.0	2.0	6.0	10.0
	**799.03**	**Sxt**	MS-WS	PLC	SA	V.Good		Subfeldspathic Arenite	5.5	41.0	46.5	*	12.0	1.8	*	0.5	0.5	*	0.5	0.2	2.8	4.0	3.2	28.0	4.0	32.0	5550.0
	**800.25**	**Sm**	MS-WS	PL	SA-SR	V.Good		Subfeldspathic Arenite	7.0	38.0	45.0	0.5	10.0	3.1	0.5	1.0	2.0	*	1.0	0.3	1.7	5.0	3.9	26.0	5.0	31.0	5559.0
	**801.00**	**Sm**	MS-WS	PL	SA-SR	V.Good		Subfeldspathic Arenite	7.0	39.5	46.5	0.5	8.0	2.2	0.5	1.5	1.5	*	0.5	0.3	2.7	5.0	3.8	25.0	7.0	32.0	6418.0
	**802.80**	**Sr**	PS	FPL	SA-SR	Poor		Feldspathic Wacke	1.0	22.0	24.0	*	15.0	3.6	2.0	1.5	32.0	*	13.0	0.4	4.1	49.5	2.4	2.0	1.0	3.0	4.0
	**803.07**	**Sm/ Sxl**	PS	PL	SA-SR	Good		Subfeldspathic Arenite	12.0	37.4	49.4	*	8.0	4.2	0.5	1.5	2.0	*	*	0.4	1.6	4.0	1.4	28.0	3.0	31.0	7069.0
	**803.57**	**Sm/ Sxl**	MS-WS	PL	SA-SR	V.Good		Subfeldspathic Arenite	8.4	34.0	42.4	1.0	12.5	1.4	0.5	1.5	3.0	*	1.5	1.0	1.0	6.5	3.2	27.0	4.0	31.0	6813.0
	**806.42**	**Sxl**	PS-MS	PLS	SA-SR	Good		Subfeldspathic Arenite	13.3	35.0	48.3	0.5	7.0	2.8	0.5	1.5	6.5	*	1.0	0.3	2.2	10.0	1.4	23.0	5.0	28.0	3858.0
	**807.20**	**Sr**	MS	FP	SA-SR	Poor		Feldspathic Wacke	4.0	20.0	24.0	*	12.5	2.2	1.5	1.0	31.0	*	21.0	0.4	1.6	54.0	2.8	1.0	1.0	2.0	4.1
**Moga 6**	**860.25**	**Sxl**	MS-PS	FPC	SR-SA	Fair-good	PBP SWP SBP	Subfeldspathic Arenite	11.6	27.8	39.4	1.5	10.5	3.2	0.4	1.6	10.8	*	1.2	0.4	1.5	13.9	4.1	22.0	3.4	22.4	3523.1
	**860.70**	**Sxt**	MS-PS	FPC	SA-SR	V.Good		Subfeldspathic Arenite	12.2	29.6	41.8	0.7	7.1	1.2	2.4	1.6	13.4	*	2.0	0.4	1.0	16.8	1.0	23.2	4.2	27.4	3422.5
	**861.20**	**Sxt**	MS	FPC	SA-A	Fair-good		Subfeldspathic Arenite	9.2	29.8	39.0	0.6	7.2	2.4	1.2	0.8	14.8	*	5.2	0.8	1.1	21.9	2.3	20.6	4.0	24.6	3434.2
	**895.39**	**Sm**	WS	CPLF	SR-SA	Good		Feldspathic Arenite	23.4	16.0	39.4	1.0	11.2	1.8	1.4	3.2	10.8	*	1.2	0.4	1.3	13.7	2.7	20.0	5.6	23.6	3434.2
	**896.00**	**Sm**	MS-WS	PCFL	SR-SA	Good		Feldspathic Arenite	14.8	24.8	39.6	1.3	12.1	1.8	1.4	0.4	8.8	*	3.4	0.4	2.0	14.6	2.4	19.0	7.4	22.4	2168.3
	**897.10**	**Sm**	MS-WS	PCFL	SR-SA	Good		Feldspathic Arenite	21.4	17.6	39.0	0.8	10.2	4.2	3.0	2.4	13.2	*	3.4	0.4	0.2	17.2	1.0	15.0	7.2	22.2	2554.4
	**899.35**	**Sm**	MS	FPL	SR-SA	Good		Feldspathic Arenite	24.4	12.8	37.2	2.0	11.4	4.2	2.8	1.6	12.4	*	1.2	0.4	1.2	15.2	3.0	15.2	7.4	22.6	2554.3
	**899.83**	**Sm**	MS	FPL	SR-SA	Fair-good		Feldspathic Arenite	24.4	16.2	40.6	0.8	8.0	3.6	6.8	1.0	6.8	*	1.4	0.8	2.1	11.1	3.3	18.4	6.4	24.8	2925.1
	**902.30**	**Sm**	MS-WS	FPL	SR-SA	V.Good		Subfeldspathic Arenite	30.4	15.8	46.2	0.4	11.4	2.2	0.4	1.2	6.4	*	*	0.4	0.3	7.1	1.1	21.2	8.8	24.0	1965.5
	**902.90**	**Fl**	PS	F	SR- SA	Poor		Feldspathic Wacke	10.6	14.2	24.8	*	6.4	4.4	1.4	1.6	41.2	*	3.6	0.1	23.6	68.4	0.2	0.5	0.5	1.0	8.2
	**906.80**	**Sm**	WS	PCL	SR-SA	V.Good		Feldspathic Arenite	16.4	24.8	41.2	2.0	12.4	2.8	0.4	0.8	8.4	*	2.4	0.8	0.5	12.1	0.9	19.4	8.0	27.4	3329.0
	**908.00**	**Sm**	WS	PCL	SR-R	Exelent		Feldspathic Arenite	14.8	24.4	39.2	1.5	14.5	2.0	0.4	1.2	7.6	*	1.2	0.8	1.1	10.7	1.5	18.8	9.2	28.0	3806.5
	**909.10**	**Sm**	MS-WS	PCLS	SR-R	V.Good		Subfeldspathic Arenite	24.0	20.2	44.2	*	12.0	2.8	0.8	2.0	6.8	*	1.0	1.0	1.5	10.3	2.1	16.8	9.2	26.0	3223.9
	**910.40**	**Sm**	MS-WS	PCL	SR-SA	V.Good		Feldspathic Arenite	19.6	18.6	38.2	2.0	15.4	4.8	0.4	2.2	9.6	*	0.8	0.4	0.6	11.4	1.0	15.8	8.8	24.6	2825.6
**Keyi 4**	**1510.55**	Sm	WS	PCL	SR-R	Good	PBP SWP SBP	Subfeldspathic Arenite	11.1	31.2	42.3	1.3	8.4	6.4	2.8	3.0	8.2	1.6	0.2	0.8	0.0	10.8	1.8	13.4	9.8	23.2	2013.4
	**1511.55**	Sm	MS-WS	PCLS	SR-R	Good		Subfeldspathic Arenite	15.8	30.6	40.4	1.0	9.2	4.4	3.6	2.0	8.6	1.2	1.4	0.8	0.0	12.0	2.0	13.0	10.4	23.4	2552.5
	**1513.50**	Sxl	WS	PCLS	SR-R	V.Good		Subfeldspathic Arenite	20.4	26.5	36.9	0.5	10.2	5.0	1.8	0.0	13.8	1.0	0.8	2.2	0.0	17.8	2.6	16.4	8.8	25.2	1994.0
	**1515.55**	FI	WS	PCLS	SR-R	V.Good		Subfeldspathic Arenite	11.0	12.3	23.3	2.7	6.4	4.4	4.2	1.8	37.6	1.2	5.8	2.0	2.0	48.6	2.4	6.4	1.8	6.2	3.0
	**1689.55**	Sxl	MS-WS	PCL	SR-R	Good		Subfeldspathic Arenite	12.0	34.6	38.6	1.2	7.6	4.4	2.8	1.6	18.6	1.2	0.4	1.2	0.0	21.4	2.8	10.8	8.8	19.6	1274.0
	**1691.40**	Sm	WS	PCL	SR-R	V.Good		Subfeldspathic Arenite	16.2	29.0	39.2	1.2	7.6	2.8	0.8	1.6	15.0	0.0	0.0	2.2	0.0	17.2	2.0	18.2	9.4	27.6	1876.0
	**1692.00**	FI	WS	PCL	SR-R	V.Good		Subfeldspathic Arenite	13.0	13.2	26.2	2.6	6.6	4.2	2.4	2.2	40.5	2.1	10.0	1.6	4.0	58.2	1.6	4.8	1.2	6.0	2.5
	**1693.00**	Sxt	MS-WS	PCL	SR-R	Good		Subfeldspathic Arenite	15.0	28.0	38.0	0.5	9.0	6.2	5.2	0.0	13.0	2.5	1.4	1.4	*	18.3	0.8	12.4	9.6	22.0	1532.2
	**1695.45**	Sm	MS-WS	PCLF	SR-SA	Good		Subfeldspathic Arenite	13.2	31.2	39.4	1.0	8.0	3.2	3.0	1.4	15.4	3.4	0.8	1.8	0.0	21.4	2.2	10.4	9.4	19.6	1271.6
**Average**									**14.1**	**25.5**	**38.4**	**1.5**	**9.7**	**3.3**	**1.8**	**1.4**	**14.2**	**1.6**	**3.1**	**0.8**	**2.1**	**20.3**	**2.2**	**16.4**	**5.9**	**21.6**	**2828.4**

WS: Well sorted; MS: Moderately sorted; PS: Poorly sorted; C: Concavo-convex; P: Point ; L: Long; F: Floating grains; S: Sutured; PBP: Primary interparticle; SWP: Secondary intraparticle; SBP: Secondary interparticle; *: Trace amount.

### Petrography

The thin section and SEM results are presented here. The primary objective of the thin section analysis is to establish mineral constituents categorize the sandstone types so as to reveal important facts about petroleum reservoir source area and paleoenvironment; paleoclimate, diagenesis and tectonic history of the area under investigation. The SEM, on the other hand, is used extensively by engineer and geologist to aid information about the pore geometry of reservoir rocks.

#### Thin-section petrography

From the glass slides, the following components of the rocks are identified: Detrital grains such as quartz, feldspar, micas (mainly muscovite and biotite), opaque and heavy minerals. Authigenic constituents like carbonate, quartz overgrowth, siderite cement, Fe oxides and authigenic clays. The minerals in addition to authigenic components are elaborated as follows.

##### Quartz

**Figure 7:**
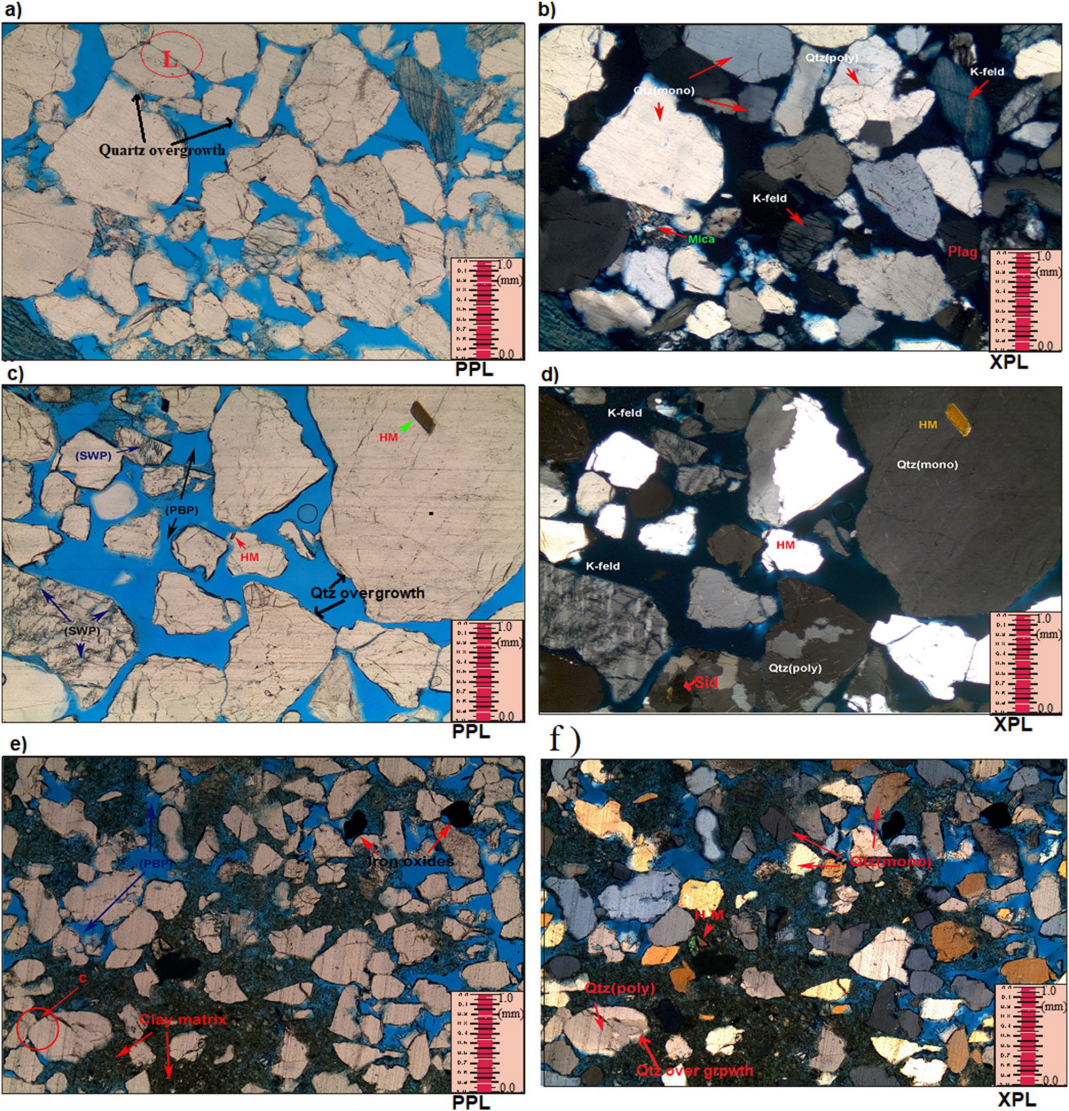
**(a**-**f**); Massive sandstone (Sm) distinguished by medium to coarse-grained sandstones (average mU-cL) with the following attributes, moderate-well sorted; sub-rounded to sub -angular; patchy cemented also moderately compacted with point; concavo-convex with few amount of long grain contacts. Common polycrystalline quartz plus monocrystalline quartz, having considerable amount of potassic feldspar (mainly orthoclase feldspar showing partial dissolution), some quantities of plagioclase, minor amount of mica as well as heavy minerals (HM), occurring as free grains (**a**-**f**); Few amounts of iron oxides occurring as pore filling, spots of siderite cement as well as lesser quartz overgrowths (euhedral crystal termination around detrital quartz grains). (**c**); Very good pore interconnectivity of primary interparticle pores (PBP, impregnated with blue dyed resin), secondary inter-particle porosity (SWP) and secondary intra-particle porosity (SBP), usually via the partial dissolution of K-feldspar. (**e**); Some patches of pore filling with detrital clays.

This is by far the most abundant in the studied sandstones. In thin sections, the quartz appears clear or colourless grains possessing a weak birefringence with a reduced refractive index that is just above that of the mounting medium^26–28^. Monocrystalline quartz (Qm) with polycrystalline (Qp) were observed in most of the samples (e.g. Fig. 7a-f). Some of the identified quartz crystals display undulose patterns of extinction. Majority of the quartz grain contains iron oxides and heavy minerals (e.g. Fig. 7a-f). The analyzed sandstones have high percentage of monocrystalline quartz, ranging from 12.3 and 41.0% (Table 5). In most of the examined samples, the polycrystalline quartz has lower percentages than the monocrystalline quartz; the polycrystalline quartz ranges in value between 5.5 and 30.4%. Most of the quartz crystals exhibit subrounded to subangular styles of roundness with some are subrounded to rounded or subangular to angular (Table 4). Besides, the quartz grains within the studied sandstones are mainly moderately sorted to well sorted (20 samples) with few well sorted (7 samples), moderate to poorly sorted (2 samples) and poorly sorted (4 samples). This might be as a result of shorter distance of transportation as well as the more abrasion experienced by the examined sandstones based on the grain shape and grain roundness.

##### Feldspars

Like quartz, feldspars in the observed thin section are clear, colourless and having low birefringence, but are distinct from quartz on account of their cleavages, twinning and refractive indices. However, differentiating between untwined orthoclase and quartz could be very challenging; but in a slide that is stained with sodium cobalt nitrate solution, the distinction between the two becomes much easier. Furthermore, feldspar grains may be decomposed partly thus appearing cloudy or turbid in contrast to the quartz grains, which invariably remain clear and unaltered^3,28^.

Among the feldspars, the K-feldspars (Fk) mainly orthoclase (Or) and perthite (Pe) are prevalent in the analyzed sandstones with relative abundances between 6.4 and 15.0% (Table 5). Plagioclase (Pl) feldspars are seen in few samples (Fig. 7a, b) with percentages between 1.4% and 6.4% (Table 5). The 2 reasons why K-feldspar is higher than plagioclase in the samples is as follows: K-feldspar is more chemically stable than plagioclase as such less stable plagioclase suffers alteration; more so, the K-feldspar minerals are more abundant than plagioclase in the continental basement rocks, i.e. the acid gneisses^29^. Hence, continental basement is considered the origin of many of the sandstones in the studied extent.

##### Micas

Platy minerals with parallel extinction identified include muscovite (Mu) and biotite (Bi) as shown in Fig. 7a, b. Muscovite appears colourless under plane polarized light displaying second order colours under crossed polar. On the other hand, biotite exhibits brown to green pleochroism masking the interference colours^27^. The amount of mica is low in all the analyzed samples. Observed biotite exists as a big detrital flakes along partings, laminae or bedding plane. The high percentage of 6.8% was recorded at a depth of 899.83 m (Table 5). Their distribution as detrital grains are controlled by sorting, and this to a large extent noticed by hydraulic behaviour of the mica flakes^29^. Also, few mica are crenulated and may exhibit bending (Fig. 7b). Noted arallel alignment of the mica flakes in the study area indicates moderate compaction.

##### Opaques

These accessory detrital components occur in trace amounts (trace amount; Table 5). Opaques are mainly hematite and pyrite with very fine—medium grained and moderate abrasions.

##### Detrital clays

Detrital clays have been recognized in all investigated samples, where their abundances vary from 0.5 to 45.0% (Table 5). The XRD analysis suggests that most of the interstitial clays are the kaolinite and chlorite (Table 6).

**Table 6:** Result of the clay minerals analysis for the analysed samples from the studied cores intervals based on XRD analysis.

Well name	Core no	Sample depth (m)	Clay minerals %				
			Kaolinite	Smectite	Illite	Chlorite	Smectite/illite
**Moga 26**	**1**	797.07	61.5	0.60	4.40	32.3	1.20
	**2**	800.25	54.2	0.00	3.80	41.1	0.90
	3	802.80	70.1	1.20	4.90	21.8	2.00
		803.07	62.3	0.30	2.80	33.2	1.40
**Moga 6**	1	860.25	47.5	0.00	0.00	52.5	0.00
		861.20	57.8	0.00	0.00	42.2	0.00
	2	895.39	80.5	0.00	0.00	19.5	0.00
		896.00	83.2	0.30	3.70	12.5	0.30
		896.35	61.0	0.00	0.00	39.0	0.00
		897.10	70.8	0.00	0.00	29.2	0.00
	**3**	899.83	69.6	0.00	0.00	30.4	0.00
	**4**	902.30	91.9	0.00	0.00	8.1	0.00
	**5**	906.80	64.3	0.00	0.00	35.7	0.00
		908.00	84.1	0.00	0.00	15.9	0.00
		909.10	82.3	0.00	0.00	17.7	0.00
		910.40	93.5	0.00	0.00	6.5	0.00
**Keyi 4**	**1**	1510.55	71.9	0.40	0.00	27.6	0.11
		1511.55	58.1	0.06	0.00	41.6	0.47
	**3**	1689.55	58.8	0.04	0.20	40.9	0.01
		1695.45	68.9	0.10	0.60	30.4	0.04
**Average**			**69.6**	**0.15**	**1.00**	**28.9**	**0.30**

##### Carbonates

These comprise mainly of siderite (Sid) and calcite forming spotty cements within the investigated samples. Occasionally, the concentration of the siderite may reach 21.0% (e.g. Table 5; well Moga 26; 807.20 m), while calcite appear only from depth 1510.55 m upward (Table 5; well Keyi 4). Siderite can be identified (under the microscope) by their brown stains along the margin of the grains towards the cleavage crack sometimes exhibiting grey or white second order interference colours (Fig. 7d).

##### Pyrite

Authigenic pyrite as cementing and replacive agent was noticed at a depth of 902.90 m (well Moga 6) where it occurs in huge amount (Table 5). However, its percentages may reach 23.6%. During the core description, pyrite nodules were recorded as cement filling within the pores. SEM analysis shows presence of patchy aggregates of sub cubic to cubic pyrite as micro crystals.

##### Quartz overgrowths

Minor amount (0.1% to 1.8%) of syntaxial quartz overgrowths were observed in all analyzed samples (Table 5). Thin sections and SEM observations revealed presence of well-developed euhedral, depilated, funnel-shaped quartz overgrowth surrounded by kaolinite.

##### Iron oxides cement

Although, iron oxide are not well developed in the investigated samples, they are present as cementing materials (e.g. Fig. 7e,f). As such, their percentages may reach 3.9% as recorded at depth 800.25 m (well Moga 26; Table 5).

#### Clay minerals analysis

**Figure 8:**
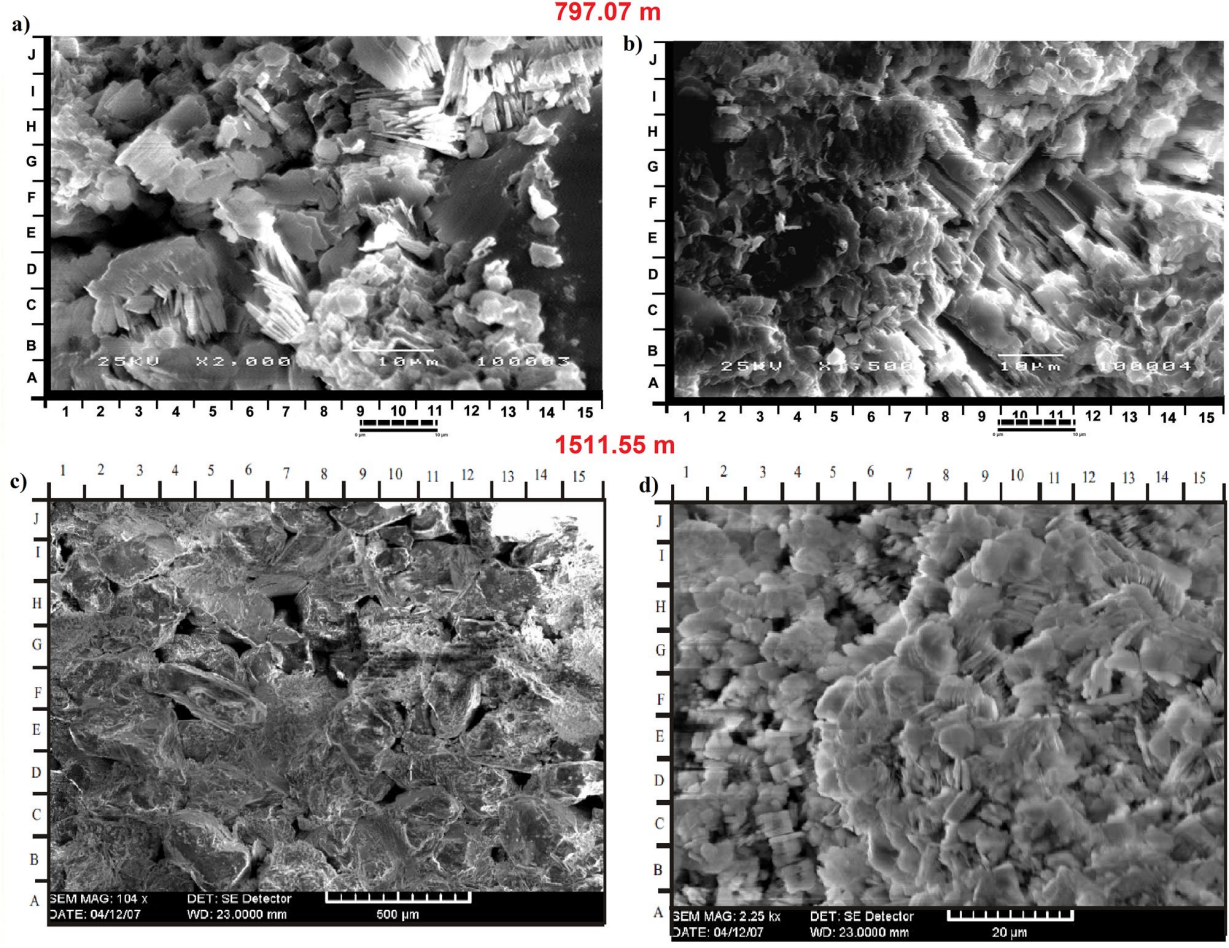
**(a-d**) SEM result showing kaolinite and chlorite minerals. Detrital plates of kaolinite partially filling some pores and partially covering some detrital grains (**a**: G9 and 11b: D14-15) which indicated as micropores. Well crystallized pore filling kaolinite booklets (**a**: G9), showing vermi-form texture (**a**: C4, G3-4, J11) of partly corroded pseudohexagonal basal plates (**a**: E-F7 and **d**: F/G 14–15) have been observed. Minor poorly crystallized ribbon-like illites (**b**, D-E12-13, F-G2) locally replace clay matrix. Some clusters of disc-like plates of chlorite were also observed (**d**: H-i 6–7).

Clay minerals are investigated using XRD and Scanning Electron Microscopy (SEM) analyses. Characteristics of each SEM sample are farther shown by two photomicrographs of the examined samples (e.g. Fig. 8). This was done to clarify the diagenetic effects of the clay minerals on the reservoir quality. Table 6 shows the XRD analysis result and is further described below:

##### Kaolinite

Kaolinite were diagnosed by 7.1 Å and 3.58 Å peaks in the dried XRD pattern which are unaltered using ethylene glycol treatment, although it was completely removed after being heated to a temperature of 550 °C^30–32^. The amount of kaolinite observed in all examined samples in amounts ranging ranges between 47.5–93.5% (Table 6). Kaolinite is formed mainly in surficial environments through a series of pedogenetic events^29^. It may also be derived from lacustrine environments as altered products of potassic feldspars under acid organic rich pore water^3,31^. More so, hydrothermally induced alteration of alumosilicate (e.g., feldspar) may still give rise to the mineral kaolinite^30^. More commonly, detrital kaolin minerals formed as a result of the effective depletion of cations and the availability of H^+^ ions^33,34^. In this case, strong leaching (which requires presence of plentiful rainfall, permeable rock and suitable topography) must be available in the source area. Thus, there would be accelerated depletion of the calcium, magnesium, sodium and potassium ions. Also, tropical and subtropical climates are essential conditions in deriving kaolinites.

The authigenic kaolinite usually form as a total alteration product of the potash feldspar in the organic rich horizons as obtainable in the studied samples at most depths. However, other clay mineral remains within analyzed intervals as detrital obtained via hydrolytic processes. Furthermore, this detrital presence of the kaolinite has been affirmed by the relatively flattened kaolinite peaks that were observed in the SEM images (Fig. 8).The kaolinite are observed as partially pores filling and partially covering some detrital grains (Fig. 8a: G9 and 8b: D14-15). Some occur as well-crystallized pore filling kaolinite booklets (Fig. 8a: G9), showing vermiform texture (Fig. 8a: C4, G3-4, J11) of partly corroded pseudohexagonal basal plates (Fig. 8a: E-F7 and d: F/G 14–15). Other detrital clay minerals occur as poorly crystallized ribbon-like illites and clay matrix replacement (Fig. 8b, D-E12-13, F-G2). Clusters of disc-like shape plates of chlorite were also observed (as in Fig. 8d: H-i 6–7).

##### Smectite

Most of the investigated samples show no (0.00%) evidence of smectite. However, for few cases of occurrence, the highest value (1.2%) was recorded at a depth of 802.80 m (well Moga 26; Table 6). It was derived during glycolation where the d-spacing of its basal reflection (001) expanded from 15 Å in the normal pattern to 18 Å in the ethylene–glycol solvated pattern^30,31^. Based on Weaver, “the climatic and topographic conditions necessary for the formation of smectite are basically opposite of those that favour the formation of kaolinite”^34^. Smectite commonly forms within low relief areas under conditions of bad drainage which will prevent rapid removal of both silica plus alkaline earth metal ions like K^+^, Na^+^, Ca^+2^ and Mg^+2^. Moreover, smectite develops normally as product of the weathering of basic and ultra-basic rocks or their metamorphic equivalences around areas of low temperatures, low precipitation and water influx. Thus, it can be inferred that we have more kaolinite than smectite.

##### Illite

Illite is non-reactive with ethylene glycol or when heated. Its identification has been based on its basal diffraction at about 10 Å. Nevertheless, illite minerals may be up to 5%^30,31^. Studied samples indicated minor quantity of illite just like the smectite mineral. Its highest concentration in this studied samples is 4.9% at a depth of 802.80 m (well Moga 26). Illite normally have more of silica, magnesium and H_2_O but less Aluminum-tetrahedral layer and low K-interlayer than muscovite according to Chamley and Moore and Reynolds^30,31^. It is principally derived from acidic igneous rocks or their metamorphic equivalence but rarely from basic rocks. Anomalous rainfall and hot climatic condition favor the formation of illite as a detrital material. Nevertheless, diagenesis favors dissolution of muscovite by illites (which is enhanced in uncluttered washed sand) while replacement of feldspar by illites instead of kaolinite is triggered by ion enrichment.

##### Chlorite

The basal spacing for structural unit of the clay mineral chlorites is close to 14 Å. Pseudo-chlorites swells up like smectite when absorbed in water or ethylene glycol, even though it is resistant to heating, where d-spacing is kept constant at 14 Å and 7 Å for the reflections 001 and 002 respectively. Amount of chlorite in the examined samples ranges between 6.5% and 52.5% (Table 6). Chlorite mineral may be regarded as a 2:1-layer group with a hydroxide interlayer, or a 2:1:1-layer group. Structurally, chlorites typically show a negatively charged trioctahedral micaceous layers in regular alternation with positively charged octahedral sheets.

Chlorites are prevalent components of low-grade metamorphism. These are less common in igneous rocks where they exist as hydrothermally driven by-products of ferromagnesian minerals. Authigenic forms of chlorites may evolve directly as by-products of the smectite via illite transformation. During the process, iron and magnesium liberated from smectite are utilized, disseminated closer and then re-precipitated together with silicon supplied from smectite or other detrital silicates^31^. Abundant chlorites within the clays are suggestive of a source rock origin that is rich ferromagnesian minerals.

## Discussion

To what degree do sedimentology, depositional processes, grain textural features, diagenetic processes and burial depth control the reservoir quality of the studied Bentiu Formation? An understanding of these processes is discussed below:

### Sedimentology and depositional processes

Obviously sedimentological analysis which include identification and characterization of the lithology, lithofacies (types/groups) and sedimentary structures play significant role in reservoir quality. Detailed facies types and groups described in “Results and interpretations” section characterized the facies associations, as well as identified the paleo-depositional environment thereby clarifying the basin evolution. This provided leads and targets for future hydrocarbon exploration.

The two main facies groups that can be identified based on composition and vertical distribution are the coarse grained and/or massive to cross-bedding sandstones (Facies Sm, Sxl, Sco, Sxt, Sh) and the fine grained/argillaceous laminated sandstones (Facies, Sr, Fm, Fl) facies group (Tables 2 and 5). The general vertical sequence, composition and internal sedimentary structures of the lithofacies in the studied core intervals suggest a deposition in a small braided channel bar/fluvial channel-filling facies association. Textural immaturity (quartz and feldspars in rich) and an overall fining-upward trend indicates a high rate of sedimentation from high-energy fluvial-origin channels sand dominated by bedload transport. Thin pebble to conglomeratic beds (usually at the base of sandy beds) were interpreted as channel lag deposits. Facies Sco/Fm with relatively thicker coarse grained (Facies Sm, Sxl, Sco, Sxt, Sh) sands being the main bar and channel deposits. The fine-grained sandy and clay-rich facies (Facies Sr, Fl), usually overlying on top of a fining-up sequence, are interpreted as overbank/channel abandonment facies deposits. It is well known that braided channel sediments make very good and productive reservoirs. Their net to gross could be higher than that of meandering reservoirs and are characterized by much less interbeddings of shale layers^35^. These form in response to rapid sedimentation with a large amount of coarse sediments influx^35^. More so, since important reservoir qualities (porosity and permeability) are usually higher in sandstone with larger grain size and lower volume of clays, hydrocarbon accumulation will be expected in the studied area^36^.

**Figure 9:**
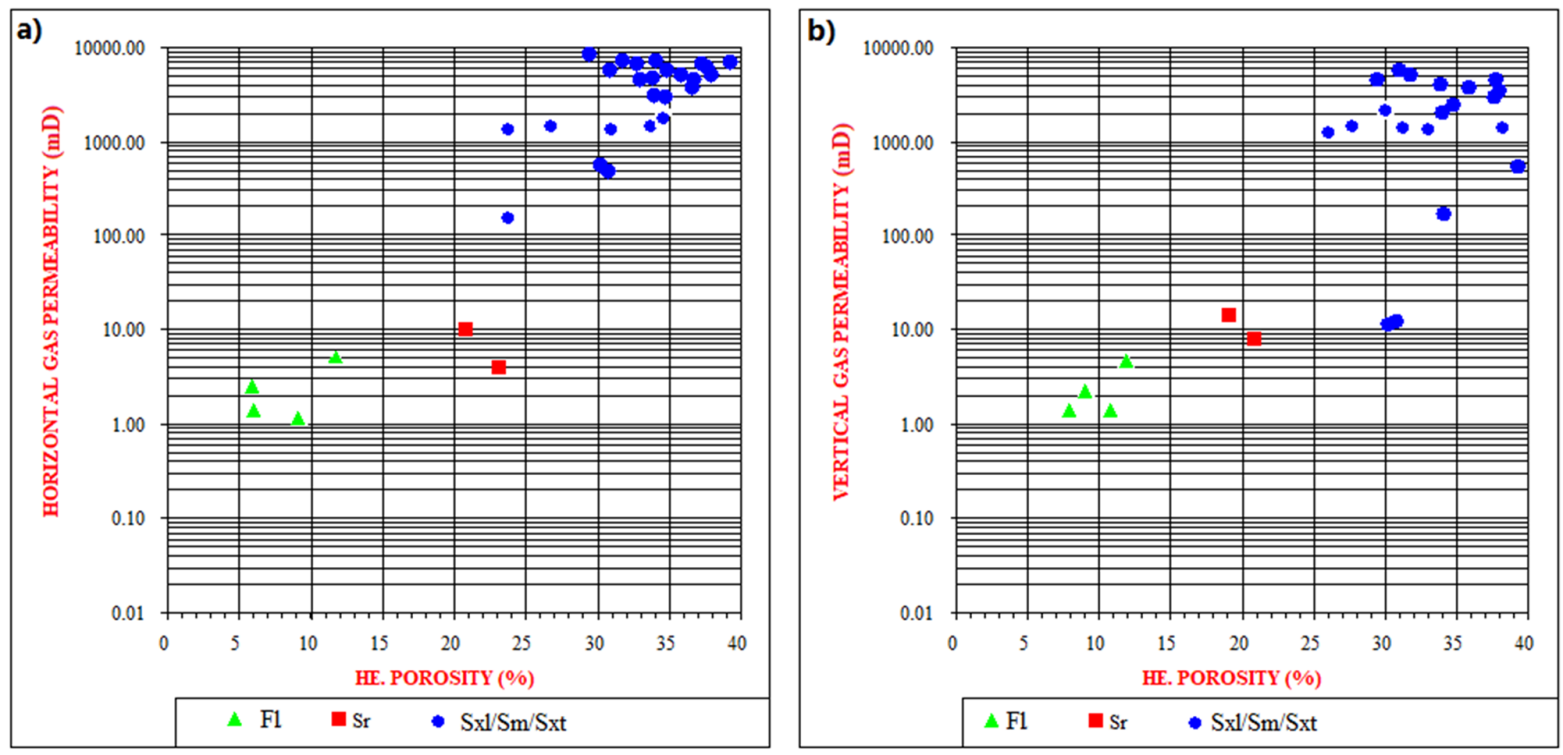
A clear relationship between reservoir quality and the various facies present in the cored intervals. The best reservoir quality is associated with the coarser grained, moderately sorted and high-energy channel sandstones (Facies Sxl, Sm, Sxt), whereas the fine and clay-rich facies (Facies Fl, Sr) display poor reservoir quality.

Considering the studied facies type as a function of reservoir potential, close examination of their porosity and permeability (Fig. 9) shows a clear relationship between reservoir quality and the various facies present in the cored intervals. The best reservoir quality is associated with the coarser grained, moderately sorted and high-energy channel sandstones (Facies Sxl, Sm, Sxt). Porosity and permeability in these facies ranges from 19.6% to 32.0% and 1825.6 mD to 8358.0 mD respectively. It has to be noted that locally low porosity and permeability in these facies is related to patchy carbonate cementation. The poorly sorted conglomeratic facies display lower permeability while the fine and clay-rich facies (Facies Fl, Sr) have poor reservoir quality. Abundant argillaceous content and extensive carbonate and clay cements have significantly reduced the porosity and permeability (as they could block pore interconnectivity) of these facies. As a result, the facies (Fl and Sr) is predominantly characterized by non-effective microporosity (2.0% to 6.0%) thus, considered non-reservoirs.

The presence of about 82% of very coarse-grained massive sandstone facies (“Results and interpretations” section; Table 2) in the studied cores is an indication of very good/excellent porosity (19.6% to 32.0%) and permeability (1825.6 mD to 8358.0 mD) zones. Other successions are composed of both sandstone and fine-grained mudstone lithofacies. The mudstone facies occur mainly at the upper part of studied cores. This is clearly shown in the fining upward sequences of the studied core-1 (well Moga 26; Fig. 5) where the sediments range from coarse-grained facies through medium grained to fine grained sandstone/mudstone. Therefore, observed relationship between the sandstone and some clay content which reduces permeability values implies that the clay content has a major control on permeability^36^. In the same way, sediments transportation process might also control the alteration in grain sizes as well as clays content in sandstones^36^. The grain sizes of the studied sandstone are composed of coarser grains being a product of very fast sedimentation rate, possibly occasioned by high-discharge events (see “Granulometric analysis” section). Therefore, a very good porosity and permeability within Bentiu Formation is anticipated. This is consistent with the grain size analysis which revealed that most of the analyzed facies samples are of coarse grain sizes with minor (3 samples) fine grain sizes.

### Textural implications

The geometrical properties (grain size, shape, sorting and packing) of sedimentary facies have direct effect on its primary porosity and permeability. Even though porosity does not depend on grain size, permeability decreases with decreasing grain sizes and the coarser the grains the higher the porosity values^3,36^. The studied area of the Bentiu Formation indicates higher content of coarse grains with very little fine to medium size grains (see “Granulometric analysis” and “Sedimentology and depositional processes” section). Similarly, the total clay content ranges from 0.5 to 14.8%, though some samples are different (31.2% to 45.0%). Facies Sxl, Sm and Sxt have more coarse grains than facies Fl and Sr (both of which have high total clay content). Also, porosity increases as the grains change from angular particles to well-rounded. Often, higher porosity values resulted from highly anisometric particles^3,27^. The studied samples exhibit grains of less variable roundness with most being sub rounded to subangular in shapes (Fig. 7a-f). Subrounded to rounded, subangular to sub rounded and subangular to angular shapes are also indicated in a few samples. Isometric particles are observed in the samples dominated by very fine grain particles and high total clay content (facies Fl and Sr). With increasing sorting, porosity and permeability are generally expected to increase. In poorly sorted sediments, smaller grains (clay and very fine grain sizes) could lead to clay plugging between the larger ones and this is known to decrease porosity and permeability^3,37,38^. The sorting of the studied sandstones is moderate to well sorted with few samples being poorly sorted (facies Fl and Sr). On the other hand, porosity and permeability decrease further by deformation and packing of grains. Loosely cemented particles usually indicate high porosity and less grain-to-grain contacts^3,38^. Most of the studied sandstone are less compacted (Fig. 7a-f). Similarly, grain-to-grain contacts are more common in the samples with higher clay content (Fl and Sr), hence has lower porosity as indicated in the samples.

### Diagenetic evolution sequence

Diagenesis may take the forms of complex interplay of processes that may occur simultaneously for a long period of time. It sometimes serves as explanations for characteristic distribution and patterns found within sedimentary reservoirs^18,39,40^.

The diagenetic features presented by the Bentiu Formation may have been influenced by compaction (mechanical and chemical), cementation (clay, quartz and carbonate), dissolution/ replacement of unstable minerals (e.g., feldspars and clays) and authigenesis (kaolinite, chlorite, Smectite/illite). This diagenetic analysis is guided by petrographical characteristics as revealed by results of thin section, XRD and SEM supported by knowledge of the grain texture and geology of the study area. Summarily, petrographic evidence revealed subangular—subrounded grains e.g. detrital quartz that indicate dominance of early compaction before cementation (Fig. 7a-f). Also, there is abundance of detrital grains showing dominance of nonlinear with few linear contacts in the samples. Furthermore, an assemblage typical of early diagenetic events can be identified by the common occurrence of few quartz overgrowths, some detrital kaolinite, chlorite and smectite in analysed samples. Most often, carbonate, pyrite and low amount of Fe oxide cements are found surrounded by detrital quartz with partial contacts (Fig. 7e,f). XRD analysis indicates presence of authigenics kaolinite, illite, smectite and chlorite. The illite and pyrite cements are replacive. In this area, there is possibility of late stage compaction (pressure solution) as temperature increases with depth of burial which will definitely lead to early chemical compaction. Early dissolution affects unstable minerals like feldspars and clay minerals. Occurrence of few linearized, concavo-convex grain contacts suggest pressure solution process or compaction/dissolution at intermediate burial stage. The studied samples display few quartz overgrowths enclosed by kaolinite and sometimes bulge out into macropores (see Fig. 7c&f). Such is an indication that quartz overgrowth is older than the enclosing kaolinite suggesting mesodiagenetic stage during which quartz cements are resistant to dissolution^41,42^. Hence, diagenetic features in the Betiu Formation sandstones are considered to have experienced complex eodiagenetic and shallow mesodiagenetic stages. This sequence is consistent with the diagenetic alteration models presented in^43–46^.

Detail XRD analysis shows 4 varieties of clay mineral constituents (kaolinite, illite, smectite and chlorite). Chlorite and kaolinite exhibit two main styles: detrital and authigenic mode of occurrence. Chlorite is widely distributed across studied samples occurring as disc-shaped clusters, pore-fillers (authigenics) and sometimes found around detrital grains as casts. The detrital kaolinite is identified by its layered style, while the authigenic kaolinite are those that partially fill available pores or encloses quartz mineral grains^18^. As a distinguishing feature, the authigenic kaolinite exhibit vermiform textures.

Smectite occurrence in the studied section is very low or zero in amounts. The Albian–Turonian period in Sudan coincided with rifting activities associated with warm paleographic conditions^18,44^. It has been reported that smectite formation in fluvio-lacustrine facies in arid/semiarid climate may be high under conditions of limited meteoric water flux^44^. The low amount recorded in the studied samples might indicate preferential kaolinite formation over smectite under conditions of high relief and episodically high rainfall that characterised the study area.

Illite also occur in minor amount as poorly developed, ribbon-like crystals in the studied section (Table 5). Its zone of anomalous occurrence is associated with significant secondary porosity of relatively elevated quartz overgrowth which may indicate mesodiagenetic stage. Smectite–illite transformation occurs in minor amount but there appears to be some correlation between it, increased chlorite and reduced kaolinite levels toward the deeper parts of the studied section (Table 6).

Dominant cements include carbonates, pyrite and iron oxides that are commonly found filling pores in disaggregated forms. The carbonates (calcite and siderite) are generally low as calcite shows patchy/spotty crystals that are restricted to deeper sections, while siderite occurs as spotty granules. Pyrites are rare, but have anomalous high amounts at a depth of 902.90 m (Table 5). The pyrites are pore-filling replacive subcubic–cubic rhombs which is an indicator of reducing conditions. Thus, the cements indicate post-compactional stage.

**Figure 10:**
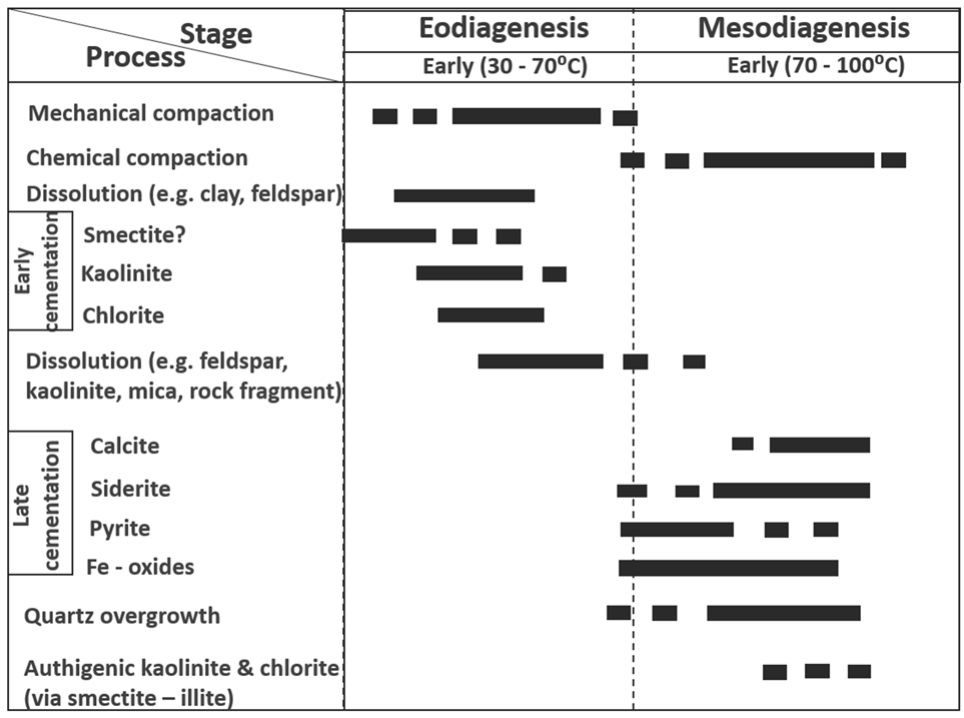
Paragenetic sequence of diagenesis in the studied Bentiu Formation. Note: the sequence here follows boundaries discussed in^44,46^ using CorelDRAW Graphics Suite 2018 v20.0.0.633 https://www.corel.com/cn; the temperature data indicated are interpretive and such should be treated with caution.

Based on evidence from the diagenetic and metasomatic features, distribution and pore-filling styles recorded in this study, the following complex paragenetic sequence (eodiagenesis—shallow mesodiagenesis) is interpreted for the evolution of the Bentiu Formation (Fig. 10):

(I) The abundance of detrital grains with nonlinear contacts (plus few linearized ones e.g., concave-convex contact) indicate pre-cementation and moderately early mechanical compaction.
(II) Detrital chlorite and kaolinite are considered contemporaneous in evolution. The high amount of chlorite is considered as primary since no indication is found post-dating the compaction. Early chlorite cements may have resulted from dissolution of feldspar or clay minerals; probably similar in evolution to kaolinite. The abundance of chlorite and the high values of preserved porosities in the studied samples are indicative of early diagenesis. Meaning that, chlorite may have acted as coating to improve grain compaction and prevent early precipitation of porosity-degrading cements like quartz overgrowth, and thus assigned eodiagenetic stage^47^. Late stage compaction (pressure solution) was experienced as temperature increases with depth of burial leading to early chemical compaction. The early dissolution affects unstable minerals like feldspars and clay minerals.
(III) Authigenic kaolinite are found partially-filling pores and enclosing quartz mineral, while authigenic quartz overgrowths are surrounded by both quartz grains and the kaolinite. This is an indication that the quartz overgrowth post-dates both the quartz grain and the early cemented kaolinite. The authigenic kaolinite is thought to have been sourced from earlier dissolution processes involving mica (kaolinite)^18,45^. The quartz overgrowth, with its observed low amounts and negligible impact on porosity and permeability in the samples, is interpreted as eodiagenetic-shallow burial. Quartz overgrowth is considered a temperature-dependent event^44,45^.
(IV) Authigenic chlorite probably happened later in the paragenetic sequence probably simultaneous or post-dating the alteration of smectite and illite. This is evident from the low amounts of smectite and illite (Table 6). Illites coincide with zones of low porosity. Observed high amounts of chlorite may be signs that it was derived from ferromagnesian minerals. Although, the chlorite may have developed via smectite—illite transformation, increasing chlorite coincides with reduced kaolinite levels toward deeper parts of the studied section. Usually, chlorite is precipitated in relation to low content of Fe and Mg in the carbonates which is formed at shallow level (> 3 km)^43^, so, chlorite in this study is placed in eodiagenetic—mesodiagenetic stage.
(V) Low and vertically restricted amount of calcite and its pore-filling nature is suggestive of cementation that is earlier than or simultaneous with chemical compaction (Table 5). The weak and restricted appearance of calcite cement as well as generally high interconnected pores indicates eodiagenetic stage event^45^. However, the high clay matrix and reduced porosities/permeabilities coinciding with siderite cements is suggestive of late stage pore-filling/replacive cementation. Hence, studied carbonate cementation is assigned late eodiagenetic—shallow mesodiagenetic stage, as lack of evidence of dissolved calcite rules out a late burial stage^45^.

Therefore, the paragenetic sequence (earliest to latest) of the Bentiu Formation is hypothesised thus: (1) mechanical compaction, early dissolution (feldspars and unstable clay minerals) (2) early cementation (involving chlorite, kaolinite and smectite) (3) chemical compaction (dissolution/alteration of clay, smectite and illite) (4) late cementation/precipitation (calcite, siderite, quartz overgrowth, Fe oxides and pyrite) and (5) authigenic chlorite and kaolinite formation.

Chlorite enrichment especially in micaceous and folded rocks is commonly associated with later stage of diagenesis^40^. This is a similar interpretation to that of the Frio Sandstones of Texas Coast^41^where the occurrence of kaolinite cements have been assigned mesodiagenetic. Also, in the Boipeba Sandstones, Reconcavo Basin, Brazil, the occurrence of kaolinite in the form of vermicules and booklet is regarded as telogenetic feature^45^, but in such case, the kaolinite will post-date quartz overgrowth cement. The possibility of paragenetic sequence assuming a complex interplay of processes that may occur simultaneously over a long period of time is sometimes used as explanations for characteristic distribution and patterns found within sedimentary reservoirs^40,47^. Assuming the various diagenetic stages occurred simultaneously, there is tendency of variations as observed in the studied samples is possible.

#### Diagenetic model

**Figure 11:**
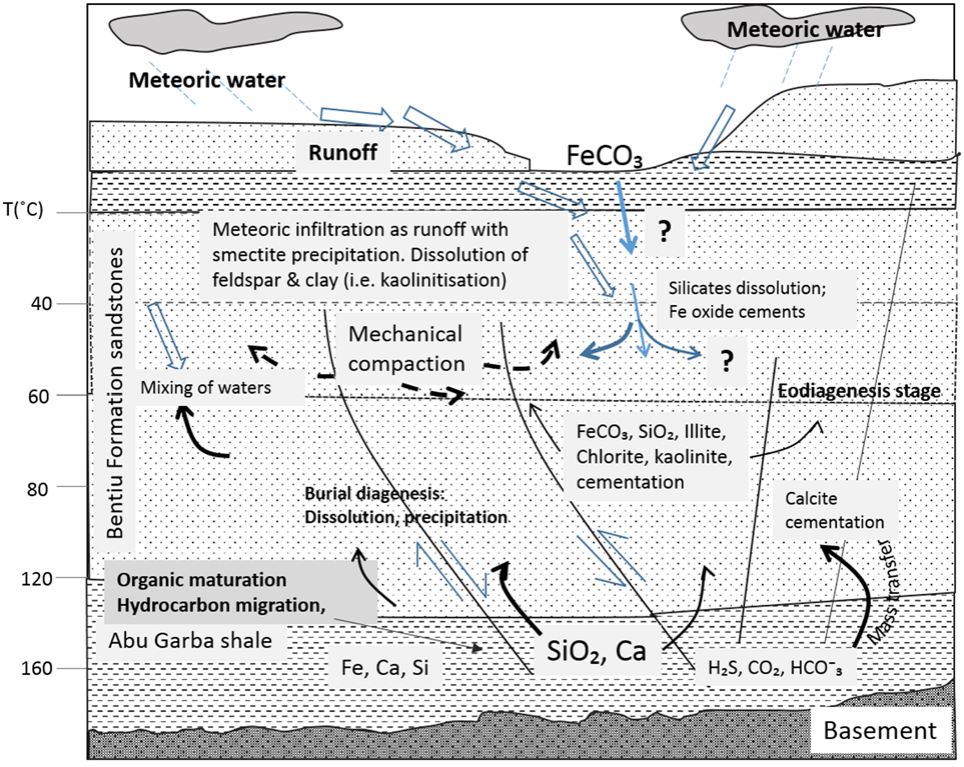
Diagenetic model for the Bentiu Formation. The Basin experienced eodiagenetic and shallow mesodiagenetic stages. Episodic meteoric water discharge causes little smetitic infiltration through porous paths, dissolution that gives rise to kaolinite. Also the organic-rich shale dissolution and hydrocarbon generation and migration at deeper depths mobilised silica, calcium and sulphide were precipitated to generate secondary porosity in the process. Note: temperature values are interpretive based on empirical studies^44,45^ using CorelDRAW Graphics Suite 2018 v20.0.0.633 https://www.corel.com/cn.

A diagenetic model is hereby proposed. The studied section of the Bentiu Formation represents surface—shallow subsurface diagenesis. It started with infiltration of clay-rich water through vadose zone and/or alteration of feldspars and clay coating. Then, meteoric recharge, breakdown of detrital K-feldspar and smectite layers in illite/smectite to convert other clay minerals (e.g., kaolinite and chlorite) as well as silica and carbonate supplied by the breaking down of deeper buried organic-rich shales and migration of hydrocarbons (Fig. 11). This model is consistent with^41,42^. Coats can decrease permeability by blocking pore throat^48^. Nonetheless, in this case, clay coating in conjunction with microquartz may have prevented early cementation and helped preserve permeability.

The Bentiu Formation diagenesis is considered to have been driven by “convective force” plus meteoric recharge. That is, an early thermal convection between high pressured underlying shales of Abu Gabra Formation and the meteoric waters (responsible for mass transfer/hydro-force) needed for the observed diagenetic changes^43,49,50^. This model is similar to the paragenetic sequence reported of the Frio Formation along the lower portion of Texas Coast^51^. The Frio Formation is underlain by the Wilcox Sandstone (Early Tertiary) with feldspathic and quartz-rich detrital grains derived from igneous rocks of intermediate composition and overlain by the Plio–Pleitocene sandstones. The Frio Formation is rich in smectite and kaolinite but less in chlorite and has paragenetic sequence that includes calcite–quartz overgrowth–calcite–dissolution kaolinite–Fe-carbonate^52^. This is similar to that of the Bentiu Formation diagenetic sequence stated above.

In this case, emphasis is only on the main processes controlling diagenesis of the studied sandstones. The diagenesis of the Bentiu Formation appears to have been mainly influenced by the sedimentary facies, climate, hydrocarbon generation and migration. The Bentiu Formation comprises of fluvio-lacustrine sandy facies that are rich in quartz (mainly microcrystalline quartz), feldspars, micas (muscovite and biotite) and widespread occurrence of kaolinite and chlorite, but low smectite. The sandstones exhibit well-sorted grains with preserved total porosity reaching 32%. That is about 80% preservation in maximum case when compared with average initial porosity of 40% and 26% preserved porosity after physical compaction at shallow level for coarse-grained sandstones^46^. Although, quartz overgrowth cements was precipitated, it has minimal effect on porosity and permeability of the sandstones. Similar minimal porosity–permeability impacts were also presented by the occurrence of calcite and siderite. Also, secondary porosities were generated in some parts of the succession.

The Moga 26 samples, at shallow depths, exhibit higher porosities while the deeper Moga 6 and Keyi 4 samples show reduced total porosity. This is an indication of inverse relationship between the depth and porosity values. Observed anomalously low porosities correspond to elevated K^+^ feldspars, siderite and pyrite cements and extremely reduced permeability values. The high value of the preserved porosity in the Bentiu Formation is consistent with the high paleo-relief suggested for the Bentiu Formation. Ordinarily, continental fluvio-lacustrine facies in warm semiarid/arid climate are expected to be rich in smectite and oxides of iron due to limited meteoric water influx^44^. But the initially high porosity of the sandstone coupled with the high relief in the area favours easier penetration of sporadic meteoric waters and dissolution of unstable detrital components. That is suitable for the formation of early kaolinite and chlorite at the expense of smectite. More so, the microcrystalline quartz and chlorite, which are believed to act as coatings that prevent early precipitations of porosity/permeability-decreasing cements such as quartz^53^, probably played role in preserving better reservoir quality at shallow levels.

Note that calcite (as cement at burial level) started forming at 1510 m, and generally increases downward (Table 5). Also, initial formation of quartz overgrowths, siderite, pyrite and Fe oxides started at shallow depth. Higher quartz overgrowth corresponds with the zone of higher calcite cement. There is also an upper and lower zones of concentration of the quartz overgrowths, the latter zone coincides with smectite-illite transition.

For the sources of carbonate and quartz cements, it is proposed that diagenesis began with the heating of organic-rich shales of the underlying Abu Gabra Formation (see Fig. 11). Breakdown/decarboxylation of organic matter leads to formation of organic carboxylic acid, CO_2_ and silica derived from maturation of organic matter and smectite—illite conversion. These processes provided the fluid used in the upward mobilizing of the components of the carbonates and quartz cements. This happened at about 80–100 °C^43^ or 80–140 °C^54^. The organic acid contributes to reduction in stability of carbonates, aluminosilicates and the discharge of silica-rich water which results in secondary porosity generation and quartz overgrowth cementation. Increased secondary porosity started at 860 m, corresponding to top of oil-stained Moga 6 and increases down gradually. However, anomalous secondary porosities were observed at 1510 m but no systematic trend is shown (as some porosity values are very high, while others are very low). This also correlate with those sections having higher calcite, siderite and pyrite cements.

Hence, the quartz overgrowth and carbonate cements were precipitated as pore-fillers. The restricted calcite occurrence is thought as the indication proximality of carbonate source. Calcium supply was exhausted in the Keyi field. CO_2_ seemed to have been released throughout. In contrast to that of the Frio Formation, the amounts of siderite is better spread in the Bentiu Formation. The water discharged by the convective force was rich in Fe, therefore, favouring siderite and Fe oxide precipitation.

Kaolinite which is the most prevalent in the middle of the studied section (Moga 6) is probably sourced from feldspar alteration. It is good to note that highest chlorite precipitation happened in Moga 26 and Keyi fields where relatively lower pyrite amounts were also observed (Table 5). Previously, it has been hypothesised that less hydrous minerals are preferentially precipitated in the presence of hydrocarbon which provides a reducing condition^46^. Hence, there is relatively higher illite with respect to smectite. Deeper in the studied section, the relative increase in cementation did not affect porosity significantly, and coincidentally, it is also the zones with the greatest secondary porosity. This is an indication of late dissolution events (probably affecting K^+^ feldspars).

The mixing of meteoric water with the silica-rich fluid favours precipitation of Fe oxides. This provides an oxidizing reaction that is consistent with the warm semi-arid setting of the area. The “convective force” plus meteoric water mass transport is supported (weakly) by the occurrence of cements at shallower levels as indicated by petrography. Alternative sources of the silica and carbonate such as marine fossils might be another possibility^43^. More data such as geochemical isotope studies is required to a better-constrained diagenetic model for the Bentiu Formation.

### Diagenetic influences

Detail petrographical analysis of the Bentiu Formation samples revealed that the sedimentary successions experienced several diagenetic episodes that impacted significantly on its porosity and permeability. Diagenesis have either resulted in a decrease or an increase in porosity and permeability (i.e. enhance or inhibit the reservoir quality)^36,39^.In the studied samples, diagenetic dissolution of feldspar and mica, partial dissolution of carbonate (calcite) cements and clays have enhanced the porosities and permeabilities. On the other hand, availability of detrital clay, kaolinite and Fe oxides precipitation, siderites and pyritic cementation, as well as compaction and quartz overgrowths have contributed to loss of porosities and permeabilities in the sediments^3,18^.

Percentages of detrital clay minerals which could reduce the porosity and permeability within the Bentiu Formation vary from 0.5 to 45.0% (Table 5). Based on XRD analysis (Table 6), kaolinites and chlorites are the main detrital clay minerals within the studied sediments. The kaolinite and chlorite can disaggregate within pores and throats, thereby decreasing porosities and permeabilities. However, the reservoir quality appears to be further enhanced by the dewatering process^36,39^. This assumption is supported by the absence or very low quantity of smectite and illite minerals observed in all the samples (see Sect. 4.4.) as well as the relatively high secondary porosity (0.5% to 10.0%) with an average of 5.9%.

Moreover, well-developed euhedral quartz overgrowth (Fig. 8a: F-G11-12) and minor microcrystalline siderite rhombs (Fig. 8b: C-D14) are also detected with rare subcubic to cubic pyrite crystals. The quartz overgrowths, pyrite and siderite are the major cementing types observed in Bentiu sandstone. Quartz overgrowth is well known as a critical factor causing reduction in reservoir quality^3,55–57^. Although some mineral cements are in low percentage, their quartz overgrowth leads to decrease in pore spaces which reduces porosity and permeability by blocking pore interconnectivity^3^. In this study, quartz overgrowth occurs in minor quantities (not exceeding 1.5%) as nucleated cells around some of the quartz grains and grow into macro pores (e.g. Fig. 7c,f), This in effect leads to decrease in the amount of macro porosity.

Generally, pyrite cement in the studied samples range from 0.2 to 3.0% with exception (23.6%) occurring at a depth of 902.90 m (see “Thin-section petrography” section). Very low values of porosity and permeability were also observed close to and around this depth (909.90 m; well Moga 6) compare to the other samples with low pyrite cement. This indicates that, pyrite cementation might have had some significant negative effect on porosity and permeability. Also affect the porosity and permeability of the analyzed samples is siderite which occurs as patchy pore-filling crystals within grains (Fig. 7d), alongside with iron oxide, is recognized as minor cements in the Bentiu Formation (e.g. Fig. 7e). Furthermore, compaction increases with burial depth because of overburden pressure and cementation of sediments leading to the reduction in porosity and permeability with increasing burial depth^3,56^. Majority of the studied coarse-grained samples are poorly compacted with absence of sutured grain network, reflecting a higher degree of compaction in coarse grained samples. However, few long grains and concavo-convex contacts occur in a few samples (e.g. Fig. 7a,e respectively). On the other hand, observed smaller intergranular pore spaces left after compaction process revealed that compaction might have had dominant control on porosity and permeability. This is consistent with the earlier discussion which indicates that sedimentology and grain textural features have a direct effect on the reservoir quality of the studied Bentiu Formation. Thus, it can be concluded that detrital clays, precipitation of kaolinite and iron oxides, cementation, by siderite and pyrite, compaction, and quartz overgrowths all have negative effect on the reservoir quality of the Bentiu Formation.

Additionally, secondary porosity is a common diagenetic process analogous to the disintegration of micas, feldspar minerals as well as calcite cement^3,58^. Both porosity and permeability can be enhanced by the dissolution of these minerals. In this study, the K-feldspar shows various degrees of alterations and dissolutions, existing as fresh uniform grains (e.g. Fig. 7a-f), partially to almost completely dissolved (skeletal) grains (Fig. 7a-d). Meanwhile, no evidence of occurrence of calcite cement in the studied samples (Table 5). Alteration and dissolution are the two processes responsible for the creation of secondary porosity in the studied Bentiu Formation sandstones. Although, these processes often result in the development of kaolinite as well as quartz overgrowth in subsequent stages. In the examined samples, kaolinite (clay) and quartz overgrowth cements have no significant effect on the reservoir quality as most of the samples have relatively high porosity and permeability (Table 5), except facies Fl and Sr with high total clay content. In support, chlorite which could improve grain compaction resistance, prevent quartz overgrowth and preserve the porosity and permeability is detected in all the studied samples^47^. Similarly, since dissolution of micas can enhance secondary porosity, therefore, sediments containing high micas contents show pressure solution compaction that damages intergranular porosity. Minor amount of micas observed in all of the investigated samples (Table 5), further supports presence of a good quality reservoir.

### Burial depth

**Figure 12:**
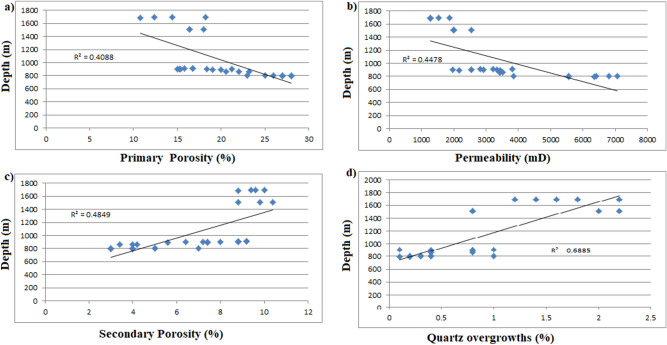
(**a**) Cross- plots of primary porosity and permeability values against depth of burial (**b**) Porosity and permeability decrease with increasing burial depth. (**c**); Secondary porosity displaying a positive trend amidst increasing burial depth. (**d**); Quantity of quartz overgrowths increasing with increasing burial depth.

**Figure 13:**
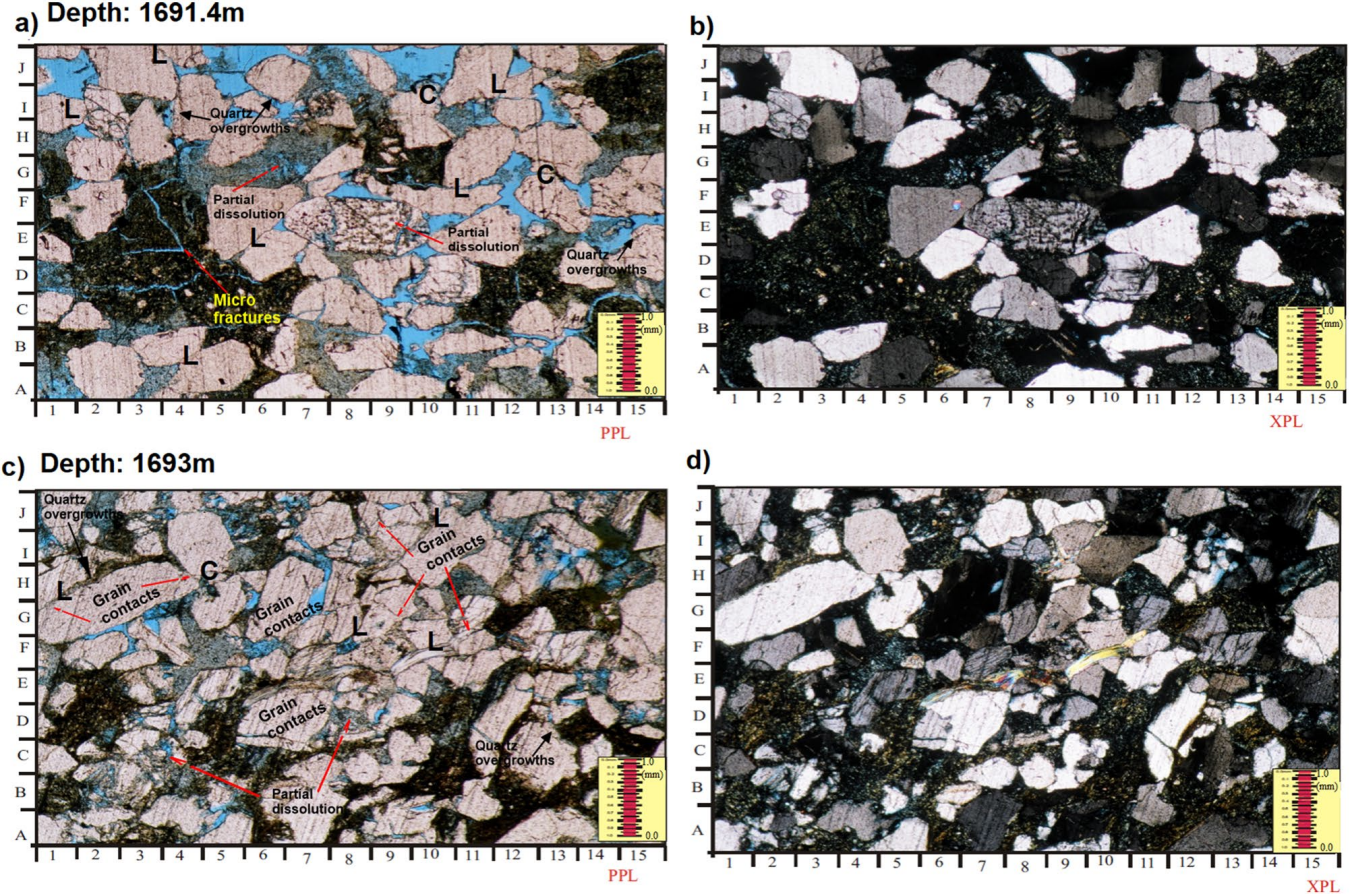
Thin section photomicrographs of medium grained (average mL-mU) sandstone sample that is well sorted, sub rounded to rounded, partially cemented with moderately to highly compacted with point, concavo-convex (photo **a** and **b**: H 5), few long (photo **a** and **b**: F-D 5–6) and fewer suture grain contacts. Mainly polycrystalline quartz (photo **b**: i-J 2–4 and photo **d**: C-E 5–7 and G-F 8–9) with more monocrystalline quartz (photo **a**: A 8–10; photo **b**: D-E 6–7; photo **c**: F–H 1–4 ) as well as considerable quantities of K-feldspars (photo **a**: E–F; photo **b**: 7–10; photos **c** and **d**: E–F 6–11), subordinate quantities of plagioclase (photo **a**: C-D 10–11). Some detrital clays occupying few pore spaces (photo **a**: G-H 4–7; photo **c**: F-G 4–5). Quartz overgrowths (photos **a**-**d**) beside some patches of iron oxides occurred as cement. Leaching and dissolution of grain, partials and micro-fractures observed in relatively high quantity (e.g. photos **a**-**d**).

The study area covers the Great Moga and Keyi oilfields, Fula Sub-Basin, Northeastern Muglad Basin. Tables 1 and 4–6 show the depth intervals and wells name of the samples. The Moga 26 and Moga 6 wells are from the Great Moga oilfield, while Keyi 4 is from the Keyi oilfield. The studied Bentiu Formation samples are of relatively shallower depths in the Great Moga (797.07 to 910.93 m) compared to the samples from the Keyi oilfield (up to 1695.70 m). From Table 5 Great Moga samples have slightly lower primary porosity and permeability than the samples from Keyi 4 well and a decreasing porosity and permeability with increasing burial depth. This is in line with the plots of primary porosity and permeability values for these samples against burial depth (Fig. 12a,b) which falls along a normal trend. On the other hand, the secondary porosity displays a positive trend with increasing burial depth (Fig. 12c). Formation of secondary porosity is usually enhanced as increase in burial depth leads to the dissolution of feldspar and other minerals as well as the dewatering of sediments^36,47^. Leaching and dissolution of grains (partial fractures and micro-fractures) are observed in relatively high quantity in the studied Keyi samples (e.g. Fig. 13a-d). Observed increase in quartz overgrowths with increasing burial depth (Fig. 12d) could be attributed to increase in temperature and pressure associated with the increase in burial depth^36^. In addition, different types of grain contact were observed at deeper depth suggesting a moderate to high compaction and porosity loss (Fig. 13a-d). Thus, the reservoir quality of the Bentiu Formation shows evidence of burial depth controls as indicated by decreasing porosity and permeability. Thus, as depth of burial increases, the compaction and cementation derived quartz overgrowths were significantly enhanced.

### Reservoir quality

The obvious connection between porosity and permeability and the variation in depositional facies controls hydrocarbon migration and accumulation. These in turn depend on grain textural features and diagenetic processes operating during or shortly after the time of deposition^59,]^. Therefore, there is the need to quantitatively determine reservoir quality using the porosity and permeability for adequate hydrocarbon accumulations^58,59^.

**Figure 14:**
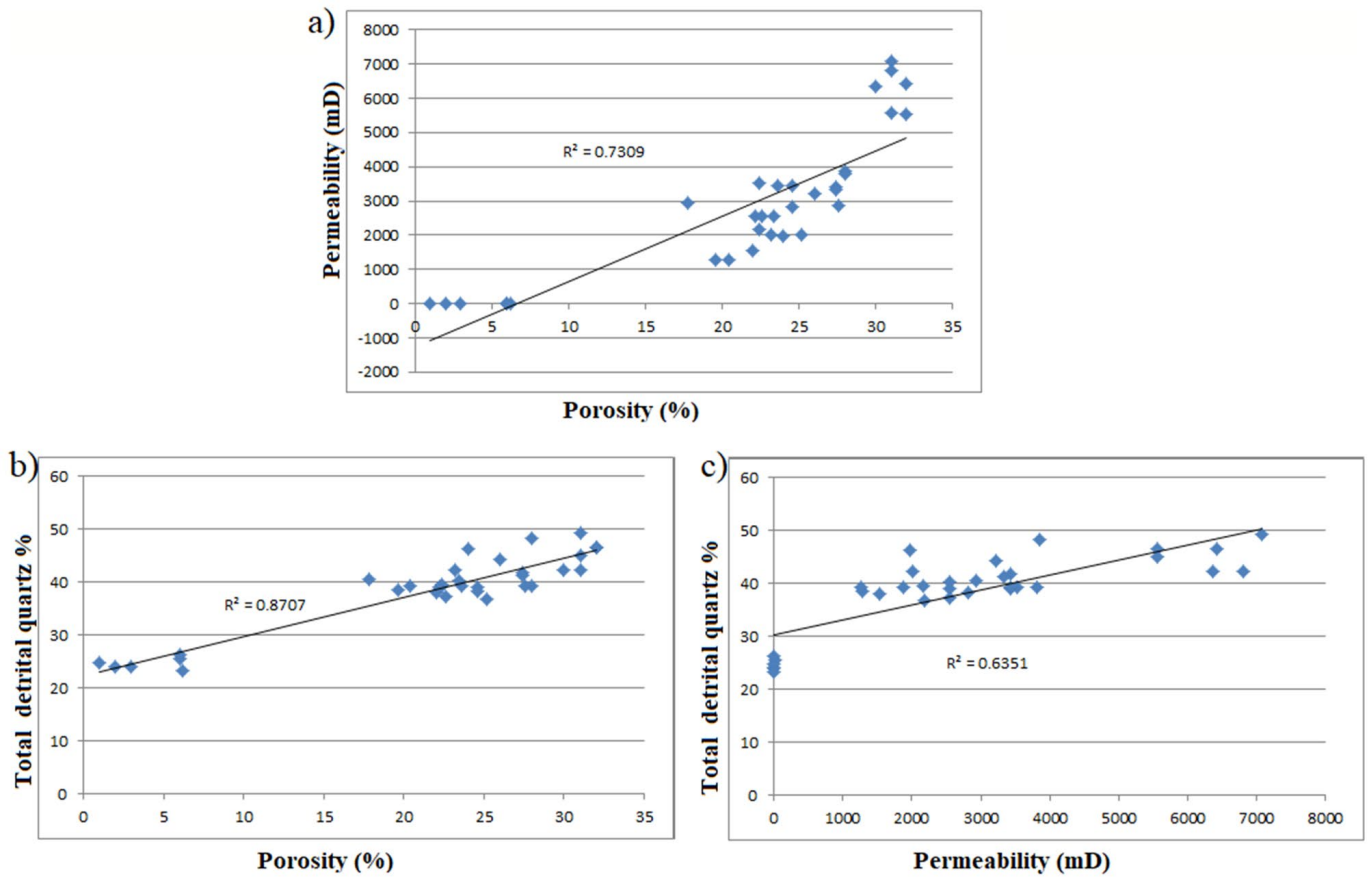
(**a**) Cross plot showing positive correlation between porosity and permeability. (**b** and **c**) Relationship among detrital quartz content with porosity and permeability respectively. The higher the detrital quartz content, the greater the porosity and permeability.

Although the porosity of the studied samples is slightly affected by the presence of some mineral cements (pyrite, siderite, clays, iron oxides and quartz overgrowths), it is nevertheless dominated by high intraparticle pores as well as considerable amount of diagenetically-related secondary porosity (partial/complete dissolution of feldspars, micas, carbonate cements and clays). Total porosity values of facies Sxl, Sm and Sxt range from 19.6 to 32.0%, whereas porosity of facies Fl and Sr ranges from 1.0 to 6.0 (Table 5). On average, pore spaces are highly variable (20–350 microns) while, the interconnected pore spaces range from poor to excellent. Absence or very weak appearance of calcite and pyrite cements helped in the preservation of relatively large pore sizes and good pore interconnectivity within the studied samples. Similarly, most of the studied samples show high permeability values. The permeability values range from 1271.6 to 7069.0 mD for facies Sxl, Sm and Sxt, while 2.5mD to 10.0mD was recorded for facies Fl and Sr (Table 5). Thus, it is inferred that facies Sxl, Sm and Sxt have very good reservoir quality, whereas facies Fl and Sr exhibit the poorest reservoir quality. Observed positive correlation between porosity and permeability suggests a close relationship; a situation where high porosity corresponds to high permeability within the studied area, if every other factors are in order (Fig. 14a).

Depositional environment is another major factor considered having a control on the grain shape, size, sorting, sedimentary structures, sand body geometry as well as diagenetic processes. Combination of these factors affects the sediments porosities and permeabilit^3,58,59^. The Bentiu Formation was deposited in a braided stream of thick sandstone beds with interbedded shale layers^3,18^. This reservoir formed as a result of rapid sedimentation where two major types of sediments were consequently deposited: (1) a large amount of coarse-grained sediments, and (2) fine grained overbank sediments. These two types of sediments have huge differences in terms of their porosity and permeability values. Thereafter, a large amount of coarse sediments was consequently deposited. The coarse grained sediments have high porosity and permeability values (19.6% to 32.0% and 1271.6 mD to 7069.0 mD) compared to the overbank sediments (1.0% to 6.0% and 2.5mD to 10.0mD) (Table 5). In addition, the coarse grained sediments are characterized by well sorted and sub-rounded to rounded grains. These characteristics account for the very good reservoir quality in the studied coarse gained sandstones compared to fine grained sediments^3,18^. This is consistent with previous studies suggesting that braided stream sediments are of larger primary porosity and better reservoir quality than the overbank deposits^3,4,10,12^. Moreover, structures and compositions of these two sediments are also different. For example, the detrital quartz content of the coarser grained sediments is higher (36.9% to 49.4%) than the fine-grained sediments (23.3% to 26.2%). The detrital quartz is more mature having higher compaction resistance which is beneficial for the preservation of primary porosity^47^. This is in line with Fig. 14b-c which shows that, the higher the detrital quartz content the greater the porosity and permeability.

**Figure 15:**
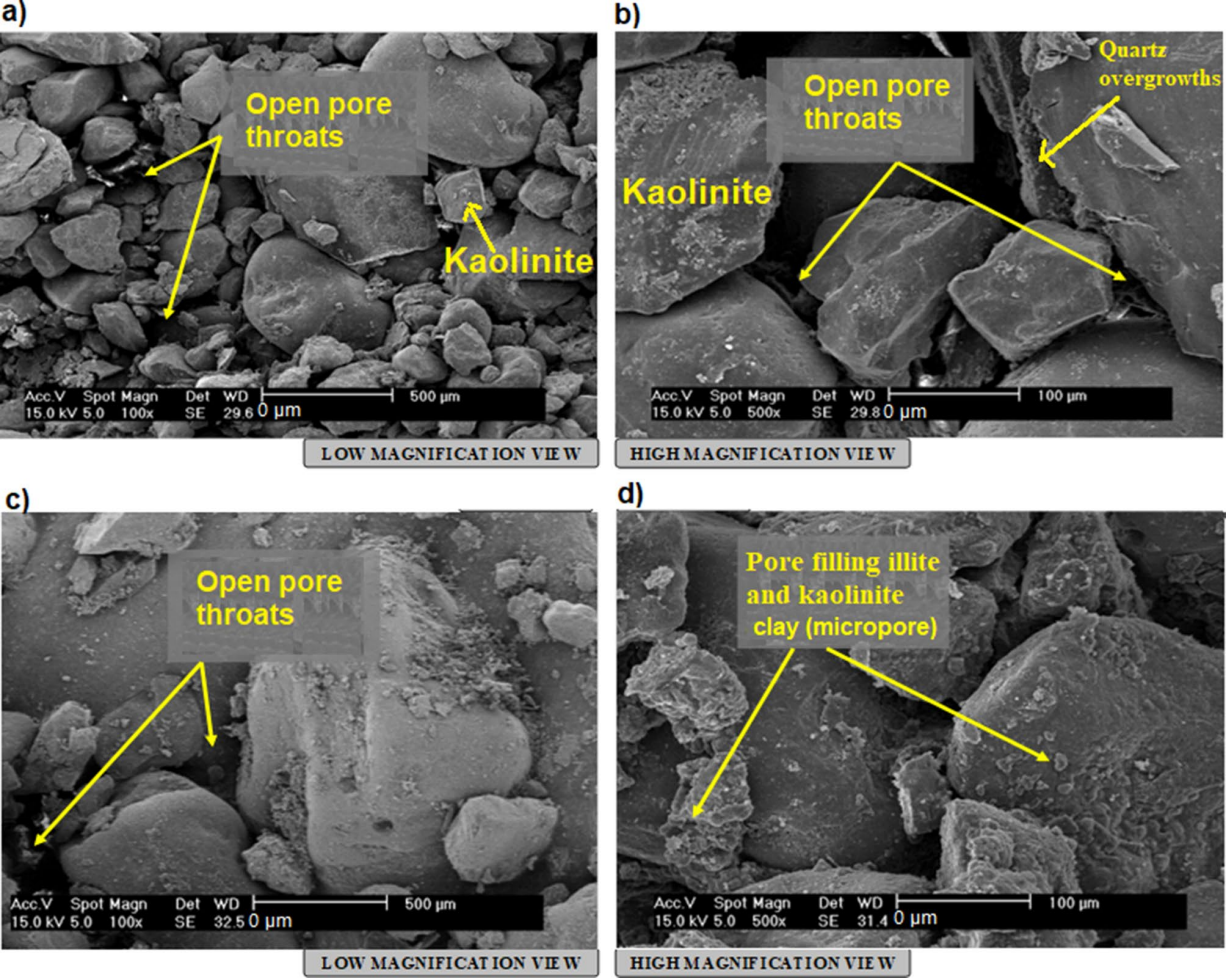
Microscopic photos obtained by SEM showing open pore throats, pore filling illite (micropores) and kaolinite as well as quartz and quartz overgrowths.

**Figure 16:**
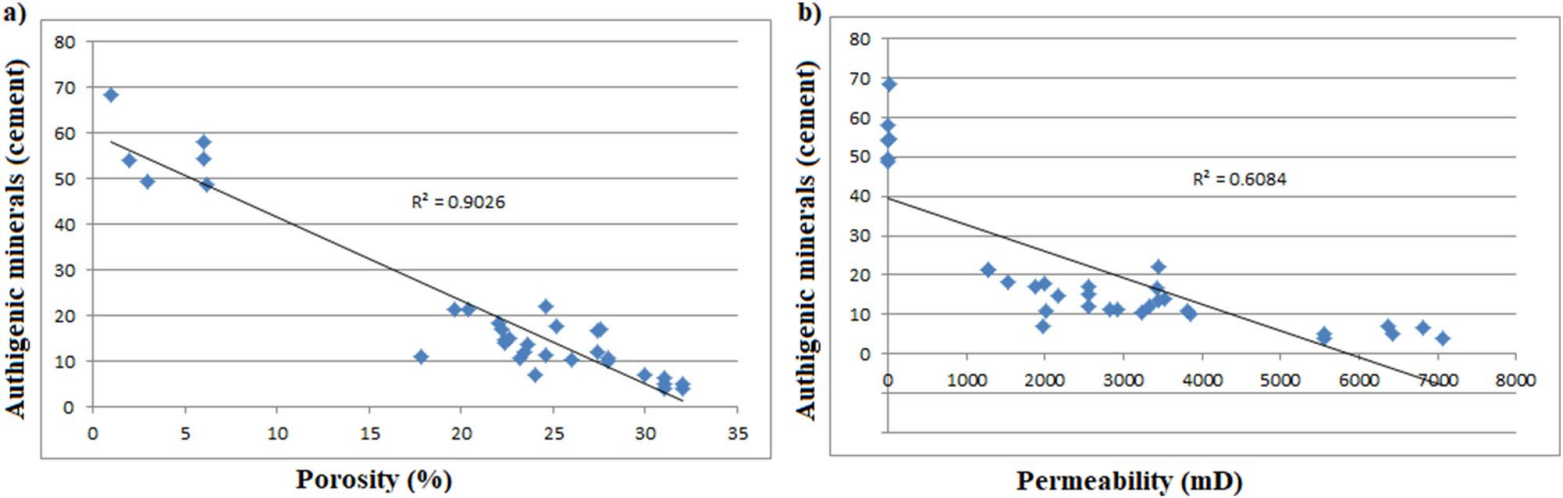
Cross-plots illustrating a good inverse correlation between authigenic mineral cements and porosity and permeability. Presence of this cements reduces the reservoir quality.

In addition to depositional environment, the reservoir quality of the Bentiu Formation is also controlled by diagenetic processes such as compaction, authigenic clays (quartz overgrowth, iron oxide, siderite and pyrite cements), dissolution of feldspar and other rock fragment. The clay minerals present in the Bentiu reservoir are mainly of chlorite and kaolinite occurring as pore filling cement. Consequently, these minerals reduce the porosity and permeability of the studied samples by blocking pore spaces (Figs. 7e, 15). Hence, samples with higher total clay content tend to have the lowest porosity and permeability values (Table 5). Similarly, siderites, pyrites and iron oxide cements also tend to block pore throats leading to reduced porosity and permeability of the samples. This makes samples with the highest cement values have the lowest the porosity and permeability values. A good inverse correlation between the sum of authigenic mineral cements and the porosity and permeability are illustrated in Fig. 16. The correlation indicates that these cements are the major concern for the reservoir quality reduction. Quartz overgrowth or quartz cementation is important in controlling reservoir quality, especially, in moderately to deeply buried reservoirs^55,56^. Quartz overgrowth developed during late diagenesis as complete or incomplete rings around the quartz grains (e.g. Figs. 7a,c, 16b). With increasing burial depth, compaction and quartz overgrowth tend to reduce the porosity by changing the grain contact from absolutely no contact to point contact or from point contact to even linear and concavo-convex contact (e.g. Figs. 7a, 12a-d). The implications is that, compaction with cementation remain the most important diagenetic processes that reduces reservoir quality with increasing burial depth. Hence it follows that the depositional regime during which sediments (especially clastics) are deposited have strong controls on shape, size, structure and type of the sediments. Furthermore, this adversely affects the primary porosity and permeability of the Bentiu Formation sandstones.

## Conclusions

In conclusion, the factors-controlling reservoir quality in the Bentiu Formation sandstone facies are: (1) depositional regime; (2) diagenetic processes; and (3) the level of sediment burial depth. Hence, porosities and permeabilities in the studied sandstones vary significantly due to these factors. Based on detailed sedimentological and petrographic analyses, the following general conclusions are made:

- The studied samples can be categorized into two main facies groups. This is established on the basis of composition and vertical distribution of the sediments as: (1) an alternating coarse to medium-grained (massive) sandstones representing a sequence of braided channel deposits, and (2) a fine grained ripple marked or laminated sandstones associated with some mudstone indicating floodplain and crevasse splay channel deposits. The coarser to medium grained sandstones contains low clay content and consequently have better porosity and permeability than the clay-rich sandstones.
- Quartz overgrowths, pyrite, siderite and iron oxide together with kaolinites and chlorites are the major cementing materials observed in Bentiu sandstones. These minerals occlude the pore throat, thereby reducing the porosity and permeability in the sandstones. However, the reservoir quality appears to be further enhanced by dissolution of feldspars, micas, carbonate cements and dewatering process. This assumption is supported by relatively high secondary porosity values (0.5% to 10.0%) with an average of 5.9%.
- With increasing depth of burial, there is obvious decrease in the porosity and permeability of the reservoirs due to effect of compaction and cementation of the studied samples.
- The reservoir quality is grossly controlled by the grain size distribution, texture and total clay content across the study area (which depends on depositional environment) as well as compaction, cementation and dissolution (with increasing burial depth).

Taking all these factors into consideration, this study can help in determining the sweet spots with higher reservoir quality within the area. Hence, the hydrocarbon exploration success can be improved and the inherent risks will be reduced.
